# ZMYND10 stabilizes intermediate chain proteins in the cytoplasmic pre-assembly of dynein arms

**DOI:** 10.1371/journal.pgen.1007316

**Published:** 2018-03-30

**Authors:** Kyeong Jee Cho, Shin Hye Noh, Soo Min Han, Won-Il Choi, Hye-Youn Kim, Seyoung Yu, Joon Suk Lee, John Hoon Rim, Min Goo Lee, Friedhelm Hildebrandt, Heon Yung Gee

**Affiliations:** 1 Department of Pharmacology, Brain Korea 21 PLUS Project for Medical Sciences, Yonsei University College of Medicine, Seoul, Republic of Korea; 2 Division of Nephrology, Boston Children’s Hospital, Harvard Medical School, Boston, MA, United States of America; Washington University School of Medicine, UNITED STATES

## Abstract

Zinc finger MYND-type-containing 10 (ZMYND10), a cytoplasmic protein expressed in ciliated cells, causes primary ciliary dyskinesia (PCD) when mutated; however, its function is poorly understood. Therefore, in this study, we examined the roles of ZMYND10 using *Zmynd10*^–/–^mice exhibiting typical PCD phenotypes, including hydrocephalus and laterality defects. In these mutants, morphology, the number of motile cilia, and the 9+2 axoneme structure were normal; however, inner and outer dynein arms (IDA and ODA, respectively) were absent. ZMYND10 interacted with ODA components and proteins, including LRRC6, DYX1C1, and C21ORF59, implicated in the cytoplasmic pre-assembly of DAs, whose levels were significantly reduced in *Zmynd10*^–/–^mice. LRRC6 and DNAI1 were more stable when co-expressed with ZYMND10 than when expressed alone. DNAI2, which did not interact with ZMYND10, was not stabilized by co-expression with ZMYND10 alone, but was stabilized by co-expression with DNAI1 and ZMYND10, suggesting that ZMYND10 stabilized DNAI1, which subsequently stabilized DNAI2. Together, these results demonstrated that ZMYND10 regulated the early stage of DA cytoplasmic pre-assembly by stabilizing DNAI1.

## Author summary

Dynein arm defects are linked to primary ciliary dyskinesia (PCD). ZMYND10 increased the stability of its interacting proteins and specifically regulated intermediate chain protein assembly, revealing tightly regulated mechanisms underlying dynein arm assembly and PCD-related pathogenesis. Increasing protein stability could be useful for developing PCD therapeutics.

## Introduction

Primary ciliary dyskinesia (PCD) is an autosomal-recessive disorder caused by defective motile cilia or flagella that is characterized by respiratory distress, impaired mucociliary clearance, chronic cough, sinusitis, bronchiectasis, male infertility, laterality defects, and cardiac anomalies in term neonates [1, 2]. To date, mutations in about 30 genes have been linked to PCD in approximately 50–70% of cases [3].

In motile cilia or flagella, the outer dynein arm (ODA) and inner dynein arm (IDA) are attached to the peripheral microtubules of the 9+2 axoneme with a fixed periodicity and generate ATP-dependent motion. Dynein arms are large, multisubunit protein complexes comprised of light, intermediate, and heavy chains [4]. The latter have ATPase activity, which provides power for the sliding between microtubules in the beating cilia. The capacity of the dynein arm to function as molecular motors depends on the integrity of its components. ODA consists of two to three heavy chains, two or more intermediate chains, and a cluster of four to eight light chains, whereas IDA has a more diverse composition [4, 5]. As such, several genes linked to PCD encode dynein arm components, including dynein axonemal light chain 1 (DNAL1), dynein axonemal intermediate chain 1 (DNAI1), DNAI2, dynein axonemal heavy chain 5 (DNAH5), and DNAH11 [6–10]. Mutations in *DNAH6*, a heavy chain of IDA, were detected in individuals with heterotaxy and ciliary dysfunction [11].

Dynein arms are pre-assembled in the cytoplasm and transported into motile cilia, where they are docked onto peripheral microtubules; however, the underlying mechanisms are poorly understood [12]. Dynein axonemal assembly factors (DNAAFs) are involved in the pre-assembly of dynein arms, and their mutation is linked to PCD [13–16]. There are five known DNAAFs—leucine-rich repeat-containing 50 (LRRC50, DNAAF1) [13], kintoun (KTU, DNAAF2) [14], DNAAF3 [15], dyslexia susceptibility 1 candidate 1 (DYX1C1, DNAAF4) [16], and HEAT repeat-containing protein 2 (HEATR2, DNAAF5) [17]. KTU and DYX1C1 interact with chaperone proteins, including heat shock protein 70 (HSP70), HSP90, and T-complex chaperonin complex [14, 16]. DNAAFs work with a chaperone complex to facilitate the proper folding of heavy chains and their assembly with intermediate chains [15]. Defects in DNAAFs result in the loss of ODAs and IDAs from axonemes [13–16].

In addition to DNAAFs, mutations in several cytoplasmic proteins, including LRRC6 [18, 19], zinc finger MYND-type-containing 10 (ZMYND10) [20, 21], chromosome 21 open reading frame 59 (C21ORF59) [22], PIH1 domain-containing 3 (PIH1D3) [23, 24], and armadillo repeat-containing 4 (ARMC4) [25], have been identified as those causing PCD when defective. Mutations in LRRC6, ZMYND10, C21ORF59, and PIH1D3 cause ODA and IDA defects [18–24], whereas those in ARMC4 cause ODA defects [25]. Thus, these proteins likely function at different stages of pre-assembly or have ODA- or IDA-specific roles. Overall, these proteins are expected to be involved in the pre-assembly of dynein arms based on their cytoplasmic localization and consequences upon loss of expression. However, the specific functions of these proteins and their relationships with DNAAFs are not well understood.

ZMYND10 (also known as BLU) has a myeloid, nervy, and DEAF-1 (MYND)-type zinc finger domain at its C-terminus that engages in protein-protein interactions [26]. ZMYND10 is highly enriched in ciliated cells compared with that in nonciliated cells [27] and is expressed in motile ciliated tissues in mice [21]. ZMYND10 has been shown to interact with LRRC6 [20, 21], although its function in motile ciliated cells is not known. Therefore, in this study, we generated and characterized *Zmynd10*^*−/−*^ mice and found that they recapitulate phenotypic aspects of human PCD, including the absence of ODA and IDA without defects in ciliogenesis or 9+2 axonemal structure. We also found that the levels of DNAI1 and DNAI2 in ODA were reduced in *Zmynd10*^*−/−*^ mice. ZYMND10 binds to and stabilizes DNAI1, thereby facilitating the assembly of intermediate chains. Our data suggested that ZMYND10 may play a role in the pre-assembly of dynein arms by regulating the expression of dynein arm components at the protein, and not the mRNA level and promoting their assembly into a cytoplasmic protein complex.

## Results

### *Zmynd10*^*−/−*^ mice recapitulated human PCD phenotypes

To investigate the function of ZMYND10, we generated mice with targeted deletion of the *Zmynd10* gene locus (S1A and S1B Fig) using a *lacZ*-containing targeting cassette (*Zmynd10*^tm1[KOMP]Wtsi^). β-Galactosidase activity staining of *Zmynd10*^*+/−*^ lung tissues on postnatal day (P)1 revealed *Zmynd10* expression in the bronchus and bronchioles, but not in the alveoli (S1C and S1D Fig). This expression pattern was consistent with that in the previous *in situ* hybridization results in mouse lung tissues [21]. *Zmynd10* expression was also observed in the testes of *Zmynd10*^*+/−*^ mice at P28 as well as in the spermatids and earlier-stage germ cells (S1E and S1F Fig). Deletion of coding exons 2–11 yielded a *Zmynd10*-null allele (*Zmynd10*^−/−^), and western blotting and immunofluorescence analyses of the testis lysates and tracheal tissue, respectively, confirmed the absence of ZMYND10 (S2 Fig).

**Fig 1:**
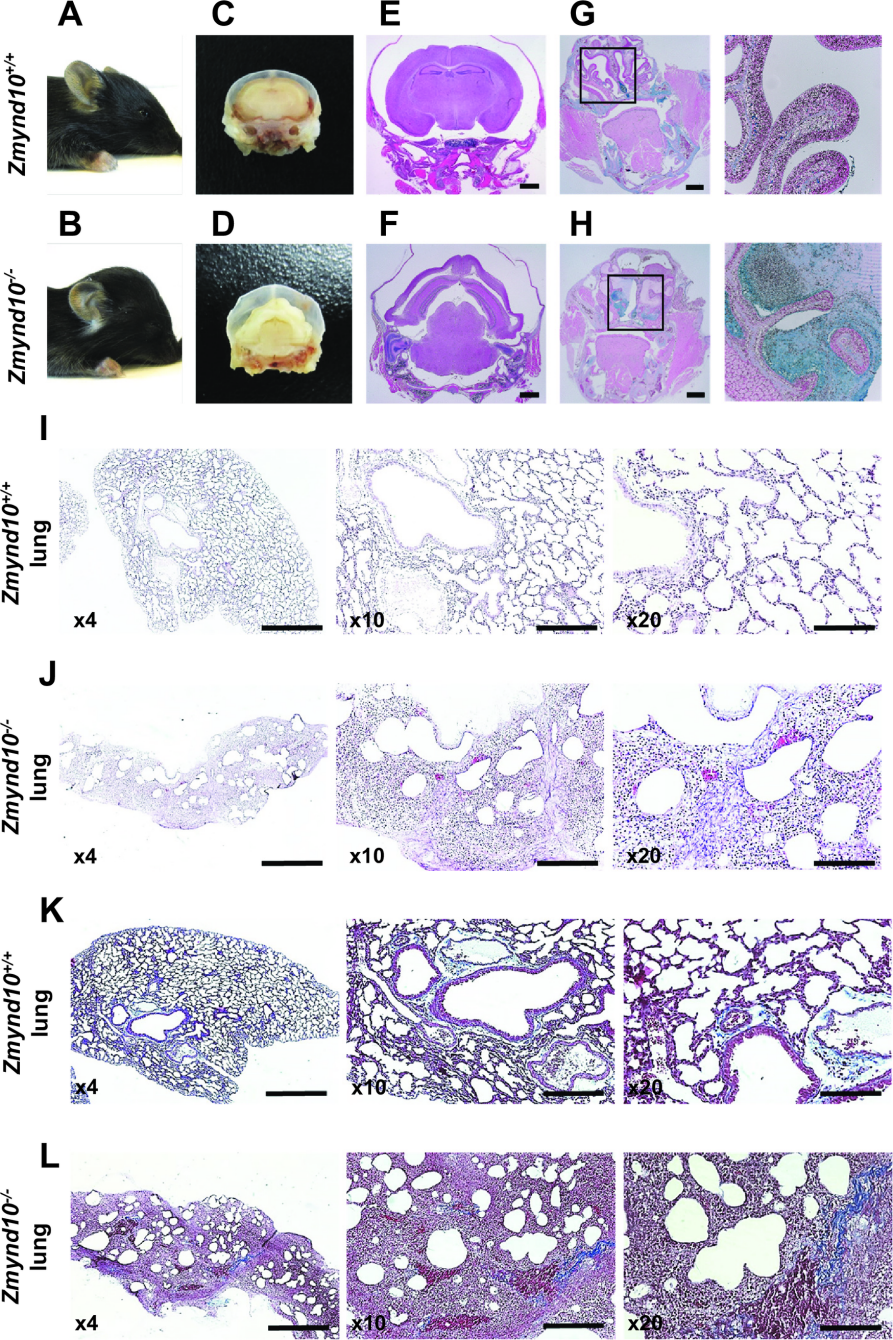
*Zmynd10*^*−/−*^ mice exhibited phenotypes consistent with cilia motility defects. **(A, B)** Unlike *Zmynd10*^*+/+*^ mice (A), *Zmynd10*^*−/−*^ mice (B) exhibited characteristic head deformation resulting from severe hydrocephalus. **(C, D)** The coronal brains sections showed enlarged ventricular cavity and reduced cortical thickness in *Zmynd10*^*−/−*^ mice. **(E, F)** Hematoxylin and eosin (H&E) staining of the coronal brain sections showed expanded ventricles in *Zmynd10*^*−/−*^ mice. Scale bars, 1 mm. **(G, H)** Alcian Blue staining of the paranasal cavities showed mucus congestion along the nasal epithelium in *Zmynd10*^*−/−*^ mice. Scale bars, 1 mm. **(I–L)** H&E staining and Masson’s trichrome staining of *Zmynd10*^*+/+*^ (I, K) and *Zmynd10*^*−/−*^ (J, L) mouse lung sections at P29 showed marked interstitial widening due to inflammatory infiltrates and edema. Original magnification, 40× (scale bar, 500 μm), 100×, and 200× (scale bar, 100 μm).

*Zmynd10*^*−/−*^ mouse litters conformed to Mendelian ratios, and neonates showed no gross abnormalities, indicating that loss of *Zmynd10* did not cause embryonic lethality; however, mutant mice exhibited growth retardation and were visibly smaller at P10, with all eventually dying within 1 month of birth (S3A–S3C Fig), with a mean survival of 14 days. *Zmynd10*^*−/−*^ mice developed hydrocephaly and subsequent abnormal head morphology, with complete penetrance (Fig 1A and 1B), and the cerebral ventricles were dilated with cortical tissue thinning (Fig 1C–1F). In addition, 42% of the *Zmynd10*^*−/−*^ mice showed laterality defects, including reversal of the heart apex, stomach, liver, or spleen (S3E and S3F Fig). Alcian Blue staining of the paranasal cavities revealed mucosal congestion in *Zmynd10*^*−/−*^ mice but not in wild-type littermates, suggesting defective mucociliary clearance (Fig 1G and 1H), which is a prominent manifestation of PCD leading to recurrent airway infection. Inflammation in the lung tissue was never observed in *Zmynd10*^*−/−*^ mice that died before P20; however, some of the mice that survived past P25 showed severe pulmonary inflammation in mutants, as evidenced by loss of alveolar architecture, thickening of alveolar septae, collapse of the alveolar space, and infiltration of inflammatory cells and fibroblasts (Fig 1I–1J and S3D Fig). Fibrosis was not observed in Masson trichrome staining (Fig 1K and 1L). Taken together, these data indicated that loss of *Zmynd10* induced defects consistent with PCD.

### Loss of *Zmynd10* caused axoneme ODA and IDA defects

**Fig 2:**
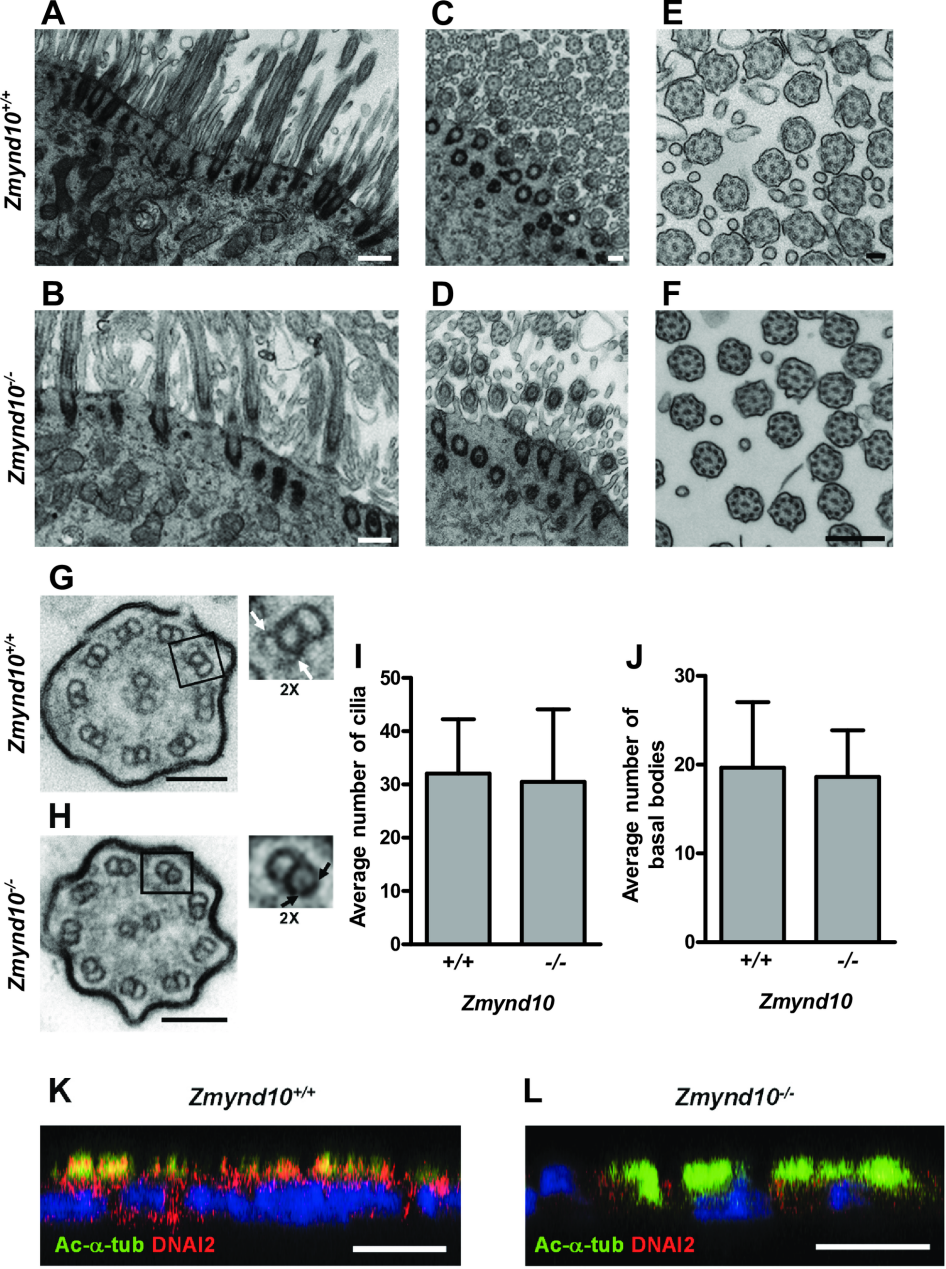
*Zmynd10*^*−/−*^ mice exhibited ODA and IDA defects. **(A–D)** TEM analysis of *Zmynd10*^*+/+*^ and *Zmynd10*^*−/−*^ multiciliated cells from the trachea of P14 mice. The number and morphology of cilia and basal bodies were similar between *Zmynd10*^*−/−*^ (B, D) and *Zmynd10*^*+/+*^ (A, C) mice. Scale bars, 500 μm. **(E, F)** TEM micrographs of the motile cilia axonemes showing normal 9+2 microtubule structures in both *Zmynd10*^*+/+*^ and *Zmynd10*^*−/−*^ mice. Scale bars, 500 μm. **(G, H)** ODAs and IDAs (white arrows) were observed in the peripheral microtubule doublets of motile cilia in *Zmynd10*^+/+^ mice (G); however, ODAs and IDAs were absent in ciliary axonemes of *Zmynd10*^*−/−*^ mice (H, black arrows). Scale bars, 500 μm. **(I, J)** Cilia (I) and basal bodies (J) were counted in TEM images of tracheas of *Zmynd10*^*+/+*^ and *Zmynd10*^*−/−*^ mice. **(K, L)** Immunofluorescence analysis of acetylated α-tubulin (Ac-α-tub, green) and DNAI2 (red) expression in mTEC cultures at ALI day 14. DNAI2 was significantly decreased and did not colocalize with acetylated α-tubulin in *Zmynd10*^*−/−*^ mTECs, suggesting that motile cilia lacked ODAs (J). Scale bars, 10 μm.

Since the *Zmynd10*^*−/−*^ phenotype suggested defects in motile cilia, we examined their ultrastructure by transmission electron microscopy (TEM). The tracheal cilia and basal bodies were abundant in the tracheal and lung epithelial cells in both *Zmynd10*^*+/+*^ and *Zmynd10*^*−/−*^ mice (Fig 2A–2D and S4 Fig); however, in the latter, some areas of the tracheal epithelium were surrounded by cellular debris and mucus (S4 Fig). Analysis of the tracheal cilia cross-sections revealed a typical 9+2 microtubular structure in both wild-type and *Zmynd10*^*−/−*^ mice (Fig 2E and 2F). In contrast, cilia in *Zmynd10*^*−/−*^ mice lacked both ODA and IDA structures (Fig 2H), which were present in the peripheral microtubules of *Zmynd10*^*+/+*^ mice (Fig 2G). This is in agreement with the previous results of studies on humans subjects with *ZMYND10* mutations who lacked ODA and IDA in the respiratory epithelium [20, 21]. The observed defects in the ciliary structure resulted in a loss of ciliary motility and beating in the ventricles of the *Zmynd10*^−/−^ brain (S1 Movie and S2 Movie). However, ciliogenesis itself was normal, as demonstrated by the observation that the numbers of cilia and basal bodies were not different between *Zmynd10*^*+/+*^ and *Zmynd10*^*−/−*^ mice (Fig 2I and 2J).

The subcellular distribution of the ODA intermediate chain protein DNAI2 in mouse tracheal epithelial cells (mTECs) cultured at the air-liquid interface (ALI) for 14 days was examined by immunofluorescence microscopy. In mTECs from *Zmynd10*^*+/+*^ mice, DNAI2 was detected in both cilia and cytoplasm and was found to colocalize with the ciliary marker acetylated α-tubulin (Fig 2K and S5A Fig). In contrast, DNAI2 was only weakly expressed in the cytoplasm of mTECs from *Zmynd10*^−/−^ mice and did not colocalize with acetylated α-tubulin, suggesting that DNAI2 was significantly decreased and failed to translocate to motile cilia in the absence of *Zmynd*10 (Fig 2L and S5B Fig). Thus, the phenotypes observed in *Zmynd10*^*−/−*^ mice resulted from a ciliary motility defect associated with the loss of axonemal ODA and IDA.

### Transcript levels of ODA and IDA components were unaltered in *Zmynd10*^*−/−*^ mice

**Fig 3:**
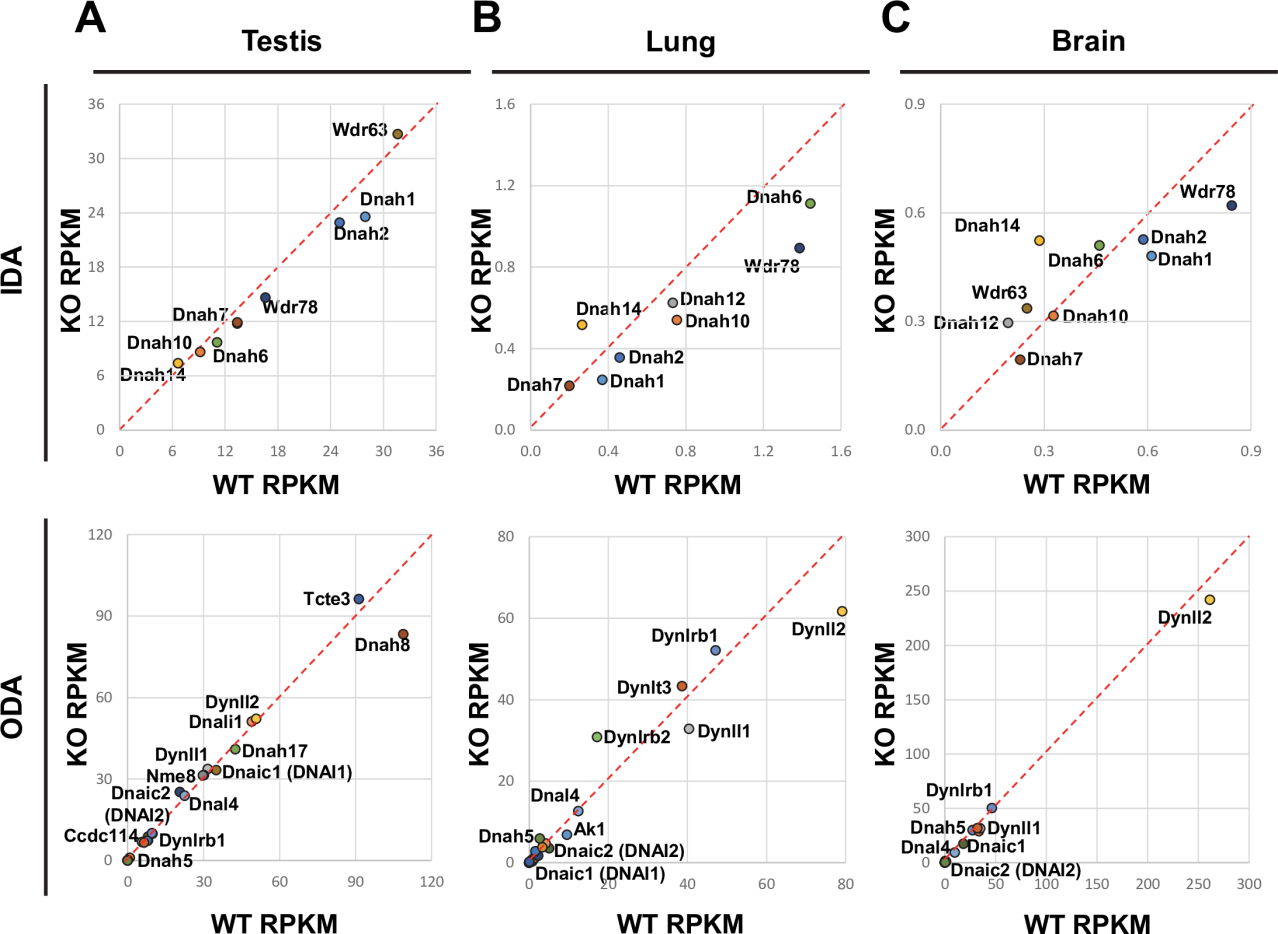
ZMYND10 did not regulate mRNA expression of dynein arm components. **(A–C)** Graphs comparing reads per kilobase of transcript per million mapped reads (RPKM) of ODA and IDA components. Total RNA was isolated from the P21 testis (A), P14 lung (B), and P14 brain (C) tissues from *Zmynd10*^*+/+*^ and *Zmynd10*^*−/−*^ mice. The expression of most dynein arm components did not differ between wild-type and mutant mice.

The mRNA levels of DNAH5 and DNALI1—ODA heavy and IDA light-intermediate chain proteins, respectively—were downregulated by ZMYND10 knockdown in cultured human tracheal epithelial cells [20]. This was examined in *Zmynd10*^*+/+*^ and *Zmynd10*^*−/−*^ mice by transcriptional profiling/RNA sequencing using total RNA extracted from the testis, lung, and brain tissues (S1 Table). The transcript levels of ODA and IDA components in mutants were similar to those in wild-type mice (Fig 3), suggesting that ZMYND10 did not regulate the transcription of dynein arm components. Gene ontology analysis showed that genes involved in muscle function were significantly upregulated, whereas those involved in ion transport were reduced in *Zmynd10*^-/-^ mice compared with those in *Zmynd10*^+/+^ mice (S6 Fig).

### ZMYND10 formed a cytoplasmic protein complex

**Fig 4:**
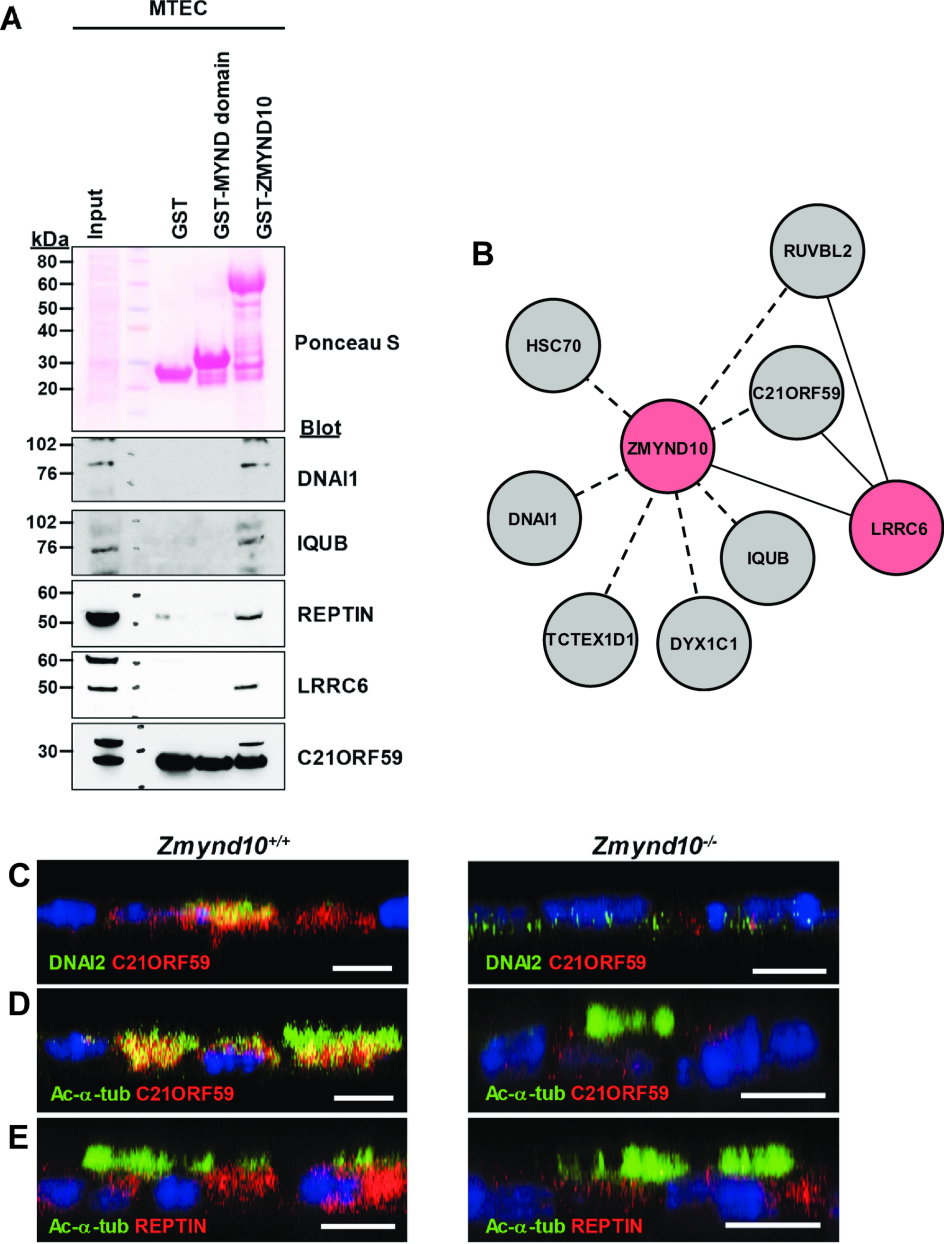
ZMYND10 formed a cytoplasmic protein complex. **(A)** GST pulldown assays of the purified MYND domain of ZMYND10 or the full-length protein. DNAI1, IQUB, REPTIN, LRRC6, and C21ORF59 in mTECs were pulled down by full-length ZMYND10, but not by the MYND domain alone. **(B)** Interactors of ZMYND10. Dotted lines are interactions identified in this study, whereas solid lines are from previous studies. **(C, D)** Immunofluorescence analysis of DNAI2 (green) or acetylated α-tubulin (Ac-α-tub, green) and C21ORF59 (red) in mTECs. At ALI day 14, C21ORF59 was observed in the cytoplasm whereas DNAI2 was localized in both cytoplasm and cilia in *Zmynd10*^*+/+*^ mTECs. DNAI2 and C21ORF59 were downregulated in *Zmynd10*-deficient mTECs. Scale bars, 10 μm. **(E)** Immunofluorescence analysis of Ac-α-tub (green) and REPTIN (red) in mTECs. REPTIN was localized in the cytoplasm of *Zmynd10*^*+/+*^ mTECs, but was absent in *Zmynd10*^*−/−*^ cells. Scale bars, 10 μm.

We and others previously reported that ZMYND10 interacts with LRRC6 [20, 21]. Given that both proteins are cytoplasmic, we examined whether they interacted with other cytoplasmic proteins that are known to be defective in PCD by co-immunoprecipitation in human embryonic kidney (HEK) 293T cells. We also obtained potential interactors from a web-based protein-protein interaction database, PrePPI (https://honiglab.c2b2.columbia.edu/PrePPI/index_old.html). ZMYND10 was found to interact with C21ORF59 and DYX1C1 (DNAAF4), IQ motif and ubiquitin domain-containing protein (IQUB), Tctex1 domain-containing D1 (TCTEX1D1), and DNAI1, but not with DNAI2 (S7 Fig). TCTEX1D1 is a dynein light chain of ODA [5], and one of the intermediate chain proteins of ODA, DNAI1, interacts with ZMYND10. Interactions with LRRC6 and REPTIN, which are essential for cilia motility [28], as well as interactions with C21ORF59, DNAI1, and IQUB were confirmed by glutathione S-transferase (GST) pulldown assays using lysates from mTECs (Fig 4A). In addition, we found that ZMYND10 interacted with heat shock cognate protein 70 (HSC70), a constitutively expressed molecular chaperone (S8 Fig), suggesting that ZMYND10 played a role in the folding and assembly of dynein arms through cooperation with HSC70. These protein-protein interactions are illustrated in Fig 4B. REPTIN and C21ORF59 expression was also examined in mTECs at ALI 14 by immunofluorescence microscopy. REPTIN and C21ORF59 signals were reduced in *Zmynd10*^*−/−*^ mTECs (Fig 4C–4E and S9 Fig). Interestingly, C21ORF59 was recently shown to interact with LRRC6 and Dishevelled (Dvl) and is implicated in planar cell polarity as well as the correct localization of ODAs to motile cilia [29]. Our results demonstrated that ZMYND10 interacted with cytoplasmic proteins associated with PCD and that some interaction partners were downregulated in the absence of *Zmynd10*.

### ZMYND10 stabilized the cytoplasmic protein complex and regulated the pre-assembly of the dynein arm in the cytoplasm

**Fig 5:**
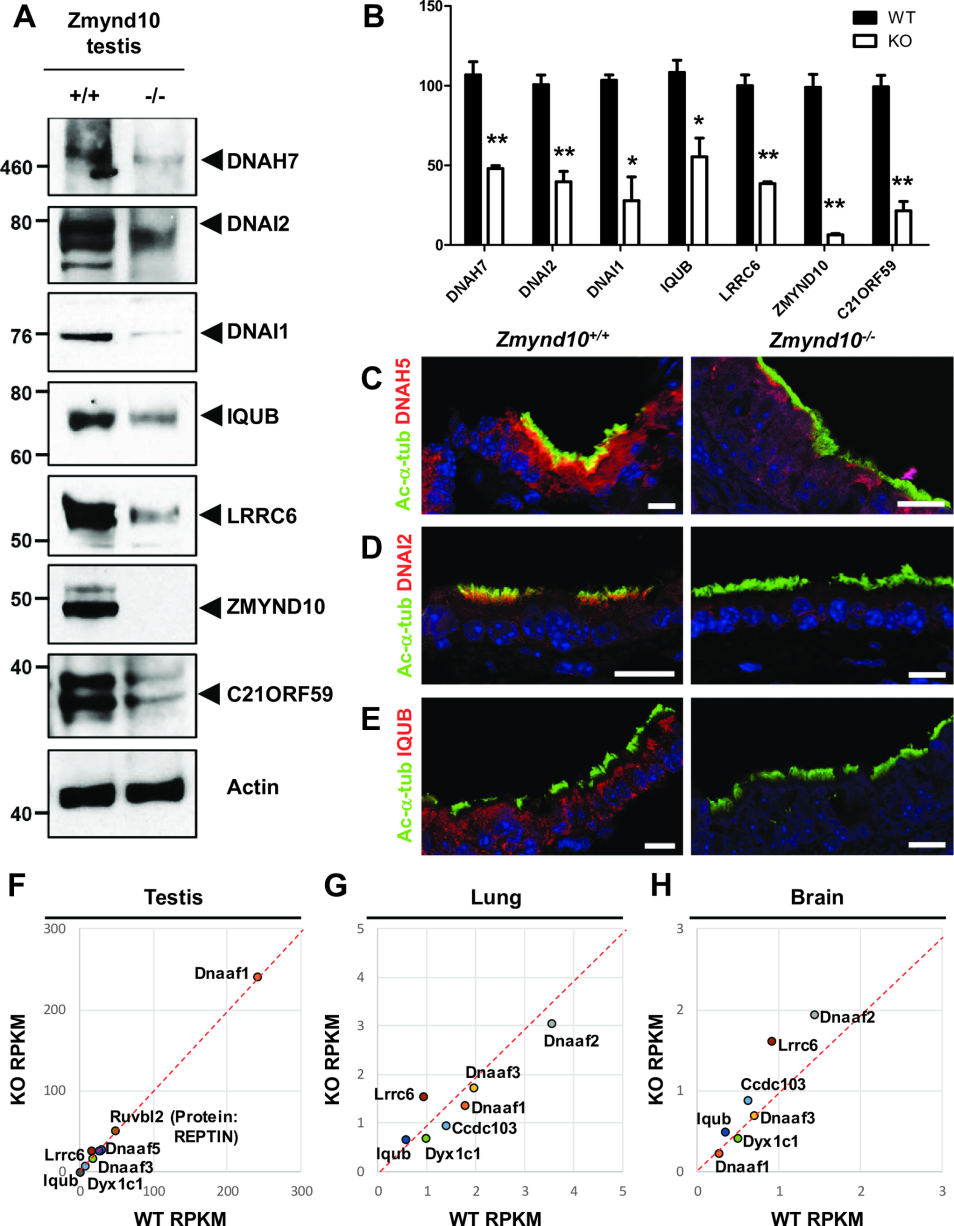
Dynein arm subunits and interaction partners of ZMYND10 were downregulated in *Zmynd10*^*−/−*^ mice. **(A)** Representative immunoblot analyses of DNAH7, DNAI2, DNAI1, IQUB, LRRC6, C21ORF59, and ZMYND10 in the testis extracts from *Zmynd10*^*+/+*^ and *Zmynd10*^*−/−*^ mice. **(B)** Bar graphs represent band intensities of the blot shown in panel A, and data represent the mean ± SD of more than three independent experiments. Band intensities were normalized to β-actin. *P < 0.05; **P < 0.005; t-test. **(C–E)** Immunofluorescence analysis of tracheal multiciliated epithelia in *Zmynd10*^*+/+*^ and *Zmynd10*^*−/−*^ mice. (C) DNAH5 (red), an ODA heavy chain protein, localized to both cilia and cytoplasm of the *Zmynd10*^*+/+*^ trachea, but was almost completely absent from the *Zmynd10*^*−/−*^ trachea. DNAI2 (red) was present in both the cilia and cytoplasm in the *Zmynd10*^*+/+*^ trachea and colocalized with acetylated α-tubulin (Ac-α-tub) along cilia. However, DNAI2 expression was negligible in the *Zmynd10*^*−/−*^ trachea (D). IQUB (red), a cytoplasmic protein and ZMYND10 interaction partner, was almost undetectable in the *Zmynd10*^*−/−*^ trachea (E). Scale bars, 10 μm. **(F–H)** Graphs comparing reads per kilobase of transcript per million mapped reads (RPKM) of dynein arm assembly factors and ZMYND10-interacting proteins in the testis (F), lung (G), and brain (H) tissues of *Zmynd10*^*+/+*^ and *Zmynd10*^*−/−*^ mice.

Our results in mTECs suggested that protein levels of dynein arm components (Fig 2K and 2L) and their interaction partners (Fig 4C–4E) were altered in *Zmynd10*^*−/−*^ mice. To investigate this in detail, immunoblotting was carried out using the testis lysates. The levels of proteins that interacted with ZMYND10, such as C21ORF59, IQUB, and LRRC6, as well as dynein arm subunits, including DNAI1, DNAI2, and DNAH7, a heavy chain of inner dynein arm, were downregulated in mutant mice (Fig 5A and 5B). This was also confirmed by immunofluorescence analysis of the mouse tracheal tissue. DNAH5, DNAI2, and IQUB signals were weaker in *Zmynd10*^*−/−*^ mice than in *Zmynd10*^*+/+*^ mice (Fig 5C–5E). A previous study in individuals with a homozygous truncating *ZMYND10* mutation showed that DNAI2 was absent from bronchial epithelial cells, whereas DNAH5 and DNALI1 remained in the cytoplasm [30]. There were no differences in the mRNA levels of dynein arm components between *Zmynd10*^*+/+*^ and *Zmynd10*^*−/−*^ mice (Fig 3). These data suggested that ODA and IDA were unstable, likely due to improper assembly in the absence of *Zmynd10*. We speculated whether the decrease in protein levels of ZMYND10-interacting factors resulted from dysregulated transcription in *Zmynd10*^*−/−*^ mice. To investigate this possibility, we examined the transcript levels of these factors and of DNAAFs, which are involved in dynein arm assembly [3], by RNA sequencing. There were no differences in the mRNA levels of these proteins between *Zmynd10*^*+/+*^ and *Zmynd10*^*−/−*^ mice (Fig 5F–5H). These results suggested that ZMYND10 stabilizes dynein arm components and their interaction partners at the protein level, but not at the transcript level.

### ZMYND10 stabilized LRRC6 and dynein intermediate chain proteins

**Fig 6:**
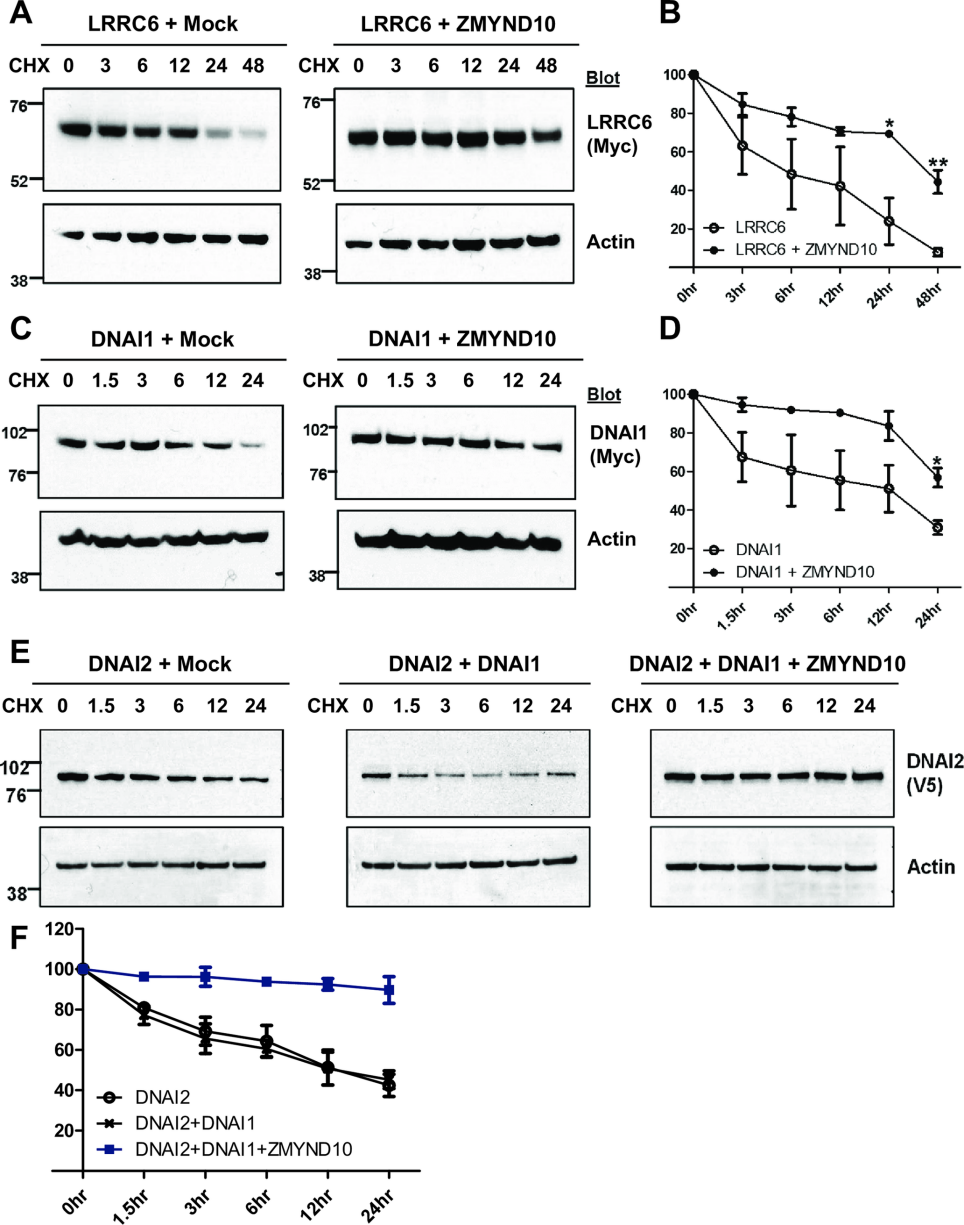
ZMYND10 stabilized LRRC6 and intermediate chain proteins of ODA. The stability of LRRC6, DNAI1, and DNAI2 proteins was examined using protein stability assays. Protein samples were harvested at the indicated times after treatment with cycloheximide (100 μg/mL).**(A, B)** Representative immunoblots of LRRC6 stability assays (A). The stability of LRRC6 was increased upon co-expression of ZMYND10 (B). **(C, D)** Representative immunoblots of DNAI1 stability assays (C). DNAI1 stability was increased by co-expression of ZMYND10 (D). **(E, F)** Representative immunoblots of DNAI2 stability assays (E). DNAI2 was stabilized by co-expression of both DNAI1 and ZMYND10 (F). Data are representative of at least three independent experiments, and band intensities were normalized to β-actin. *P < 0.05; **P < 0.005; t-test.

To determine whether ZMYND10 regulated its interaction partners and dynein arms at the protein level, we investigated the effects of ZMYND10 on the stability of LRRC6, DNAI1, and DNAI2 in a heterologous system. HEK 293T cells overexpressing LRRC6 and/or ZMYND10 were treated with cycloheximide (100 μg/mL) for up to 48 h to block protein synthesis (Fig 6A). Only 7.8% of LRRC6 remained after 48 h of treatment. However, this value was increased to 44.4% when LRRC6 was co-expressed with ZMYND10 (Fig 6B), suggesting that ZMYND10 prevented LRRC6 degradation. Similarly, the amount of DNAI1 protein was increased from 30.9% to 64.1% by co-expressing ZMYND10 (Fig 6C and 6D), but not by co-expressing ZMYND10-p.Gln366* mutant protein, which lacked the MYND domain and was identified in a PCD patient [20] (S10 Fig), indicating that the MYND domain was necessary for the stabilizing effect. In contrast, the stability of DNAI2, which did not interact with ZMYND10 (S7K and S7L Fig), was unaffected by ZMYND10 overexpression (S11 Fig). Given that both DNAI1 and DNAI2 levels were downregulated in *Zmynd10*^−/−^ mice, we speculated that ZMYND10 stabilized DNAI1, which in turn stabilized DNAI2. To evaluate this possibility, we compared the protein levels of DNAI2 upon co-expression with DNAI1 without or with ZMYND10. DNAI2 was more stable in the presence of both DNAI1 and ZMYND10 than in the presence of DNAI1 alone (Fig 6E and 6F, and S12 Fig). These results demonstrated that ZMYND10 stabilized some of its interaction partners at the protein level and modulated the pre-assembly of intermediate chains by stabilizing DNAI1.

## Discussion

In this study, we generated and characterized *Zmynd10*^*−/−*^ mice as a model for human PCD. The mice exhibited loss of ciliary motility and ODA and IDA components without disruption of ciliogenesis, thereby recapitulating the phenotypes associated with *ZMYND10* mutations in humans and serving as an appropriate model to study PCD pathogenesis.

The assembly of dynein arms into motile cilia is a complex process involving many regulatory factors, including DNAAFs and chaperones, that contribute to the stabilization, folding, and pre-assembly of dynein arm components [15, 24, 31] into multiprotein complexes that undergo intraflagellar transport into the axoneme for attachment to peripheral microtubules [31]. In *Chlamydomonas*, dynein arms exist as intermediate-chain/heavy-chain (IC-HC), light chain, and docking complexes [12]. During ODA pre-assembly, HCs, such as DNAH5 or DNAH11, are attached to ICs, DNAI1 (IC1), and DNAI2 (IC2). This process is facilitated by DNAAF1, DNAAF2, and DNAAF4, while DNAAF3 is proposed to act during the final stages of chaperone dissociation [15]. IC-HC assembly fails in the absence of the IC subunit [12]. Biochemical analyses of *Zmynd10*^*−/−*^ mice showed that DNAI1 and DNAI2 are downregulated, suggesting that ZMYND10 stabilizes these two proteins or mediates their assembly. Given that formation of the IC complex precedes IC-HC assembly, the reduced levels of DNAI1 and DNAI2 may account for the observed decrease in an ODA HC, DNAH5.

In this study, we demonstrated that ZMYND10 formed a cytoplasmic protein network comprised of LRRC6, C21ORF59, DYX1C1, IQUB, REPTIN, and HSC70. The interaction between LRRC6 and REPTIN is essential for cilia motility in zebrafish, although this function is independent of its known role as a transcriptional regulator [28]. Similarly, although ZMYND10 binds to REPTIN, the expression levels of various dynein arm components and interactors of ZMYND10 were not diminished in *Zmynd10*^*−/−*^ mice, ruling out transcriptional regulation of these factors as a mechanism underlying ODA and IDA defects. We previously showed that *DNAH5* and *DNALI1* mRNAs were downregulated in human tracheal epithelial cells in which an shRNA targeting ZMYND10 was delivered by lentivirus. This discrepancy between in vivo mouse and cell line data may be due to artefacts resulting from lentiviral vector integration [32]. C21ORF59 interacts with LRRC6, DNAAF1, and Dvl to regulate polarization as well as ciliary motility [29]. DYX1C1 interacts with KTU, HSP70, HSP90, and T-complex chaperonin [14, 16], whereas REPTIN interacts with PIH1D1 [33], which contains a PIH (protein interacting with HSP90) domain implicated in the pre-assembly of dynein arms [34]. PIH1D3 interacts with KTU, DYX1C1, and HSP90 [23, 24] and is implicated in the formation of the IC complex, as evidenced by its interaction with DNAI2 and the downregulation of DNAI1 and DNAI2 in *Pih1d3*^*−/−*^ mouse sperm [24, 35]. ZMYND10 is functionally similar to PIH1D3 in that both proteins interact with DYX1C1 and heat shock proteins; moreover, ICs are reduced in mice lacking *Zmynd10* or *Pih1d3* [35]. ZMYND10 interacts with HSC70, a member of the HSP70 family. HSC70 is involved in diverse cellular processes, including protein folding and protein degradation, and exerts its chaperone activity by cooperation with cochaperones and by binding to nascent or unfolded polypeptides through the substrate binding domain in an ATP-dependent manner [36]. Therefore, it is possible that ZYMND10 affects the stability of DNAI1 through cooperation with HSC70.

Currently, there is no curative therapy for PCD. For PCD resulting from a defective dynein arm component, the component should be replaced with a normal one. However, this will be challenging considering the huge size of some dynein components. For example, the coding region of *DNAH5*, which is most frequently mutated in PCD [17], is about 15.6 kb, encoding a protein of 529 kDa. In this regard, PCD resulting from defects in DNAAFs or other cytoplasmic proteins is different in that dynein arm components are not compromised, but their cytoplasmic assembly or trafficking is defective. In this study, we demonstrated that the protein levels of DNAI1 and DNAI2 were reduced in *Zmynd10*^*−/−*^ mice due to the decreased stability of DNAI1 in the absence of ZYMND10. Therefore, increasing protein stability of DNAI1 can be considered as a potential treatment.

In conclusion, our results demonstrated that several cytoplasmic proteins, including ZMYND10, formed a protein network in motile ciliated cells that, in conjunction with chaperone proteins, modulated various aspects of dynein arm pre-assembly. ZMYND10 specifically functioned in the early steps of this process by regulating DNAI1 stability or folding, thereby controlling IC assembly (S13 Fig). These findings provide insights into the molecular mechanisms involved in dynein arm assembly and the pathogenic basis for PCD-associated defects. This will also help to develop pharmacological interventions for PCD caused by defects in the cytoplasmic nonaxonemal components of motile cilia.

## Materials and methods

### Ethics statement

The animal protocol was reviewed and approved by the Institutional Animal Care and Use Committee of University of Michigan (#08619), Boston Children's Hospital (#13-01-2283), and Yonsei University College of Medicine (#2015–0178). All animals were handled in accordance with the Guidelines for the Care and Use of Laboratory Animals.

### Mice

Targeted *Zmynd10*^tm1(KOMP)Wtsi^ embryonic stem cells were obtained from the Knockout Mouse Project Repository and injected into blastocysts. Chimeric mice were bred with C57BL/6J mice to establish germline transmission. Wild-type littermates were used as controls for *Zmynd10*^*−/−*^ mice. Genotyping was performed by standard PCR using the primers Zmynd10-ex2F (5′-TGGAGGAGCTTGGAACTGAC-3′), Zmynd10-ex2R (5′-GGAGGCAGACACAGTTAGGC-3′), and CSD-RAF5-F (5′-ACACCTCCCCCTGAACCTGAAA-3′, SR1 (5′-TGCTTTATTGTGCGAAAGGAAGAGGG-3′).

### β-Galatosidase staining

P1 or P28 mice were sacrificed, and the testes and lungs were dissected. After three washes with phosphate-buffered saline (PBS), the tissues were fixed in 4% paraformaldehyde (PFA)/0.02% Nonidet (N)P-40 for 2 h at room temperature and permeabilized with 0.02% NP-40 in PBS for 1 h. Samples were incubated overnight at 37°C in X-gal staining solution composed of 5 mM K_3_Fe(CN)_6_, 5 mM K_4_Fe(CN)_6_, 2 mM MgCl_2_, 0.01% sodium deoxycholate, 0.02% NP-40, and 1 mg/mL X-gal in PBS. They were then washed three times with PBS for 5 min each and post-fixed with 4% PFA for 24 h before embedding within paraffin. Sections (10 μm thick) were deparaffinized and rehydrated through a graded series of ethanol concentrations followed by counterstaining with Nuclear Fast Red (Vector Laboratories, Burlingame, CA, USA).

### Histology

The lung and snout tissue specimens were fixed using 10% formalin for 24 h. The tissues were sectioned (5 μm thickness) and stained with hematoxylin and eosin, or periodic acid-Schiff for histological examination.

### TEM analysis

The tracheas of *Zmynd10*^*+/+*^ and *Zmynd10*^*−/−*^ mice at P14 were dissected and fixed using 2.5% glutaraldehyde, 1.25% PFA, and 0.03% picric acid in 0.1 M sodium cacodylate buffer (pH 7.4) overnight at 4°C. Samples were then processed for TEM analysis using standard techniques.

### Videomicroscopy of the ependymal cilia in mice

P16 mice were deeply anesthetized and then decapitated. The brain was rapidly removed and immersed in ice-cold Dulbecco’s modified Eagle’s medium (DMEM; Invitrogen, Carlsbad, CA, USA) supplemented with 10% fetal bovine serum (FBS; Sigma-Aldrich, St. Louis, MO, USA). Sagittal sections of 150-μm thickness were cut using a vibratome (VT1200S; Leica, Wetzlar, Germany). Sections from the third ventricle were visualized on an Axio Observer A1 microscope using a 63× phase contrast objective lens (LD Plan-Neofluor 0.75 Corr Ph2 M27; Carl Zeiss, Jena, Germany) equipped with a high-speed charge-coupled device camera (optiMOS sCMOS; QImaging, Surrey, BC, Canada). Movies were acquired at 100 frames/s.

### Antibodies

A polyclonal antibody recognizing the C-terminal sequence (amino acids 339–362, DRLERENKGKWQAIAKHQLQHVFS) of mouse ZMYND10 was recovered from rabbits injected with the corresponding antigen (AbFrontier, Seoul, Korea). LRRC6 and DNAH5 antibodies were previously described [37, 38]. Antibodies against DNAI2 (H00064446-M01; Abnova, Taipei, Taiwan); REPTIN (ab89942; Abcam, Cambridge, UK); DNAH7 (NBP1-93613) and DNAI1 (SAB4501181; both from Novus Biologicals, Littleton, CO, USA); IQUB (HPA020621) and TCTEX1D1 (HPA028420; both from Sigma-Aldrich); acetylated α-tubulin (T7451 from Sigma-Aldrich and 5335S from Cell Signaling Technology, Danvers, MA, USA); FLAG (#8146) and Myc (#2276; both from Cell Signaling Technology); and C21ORF59 (sc-365792) and β-actin (sc-1615; both from Santa Cruz Biotechnology) were purchased from commercial sources. Secondary antibodies were purchased from Invitrogen and Santa Cruz Biotechnology for immunofluorescence and immunoblotting analyses, respectively.

### Immunofluorescence analysis

mTECs grown on inserts were fixed using 4% PFA for 10 min and permeabilized with 0.1% Triton X-100 for 20 min at room temperature. The tracheal tissue was fixed in 4% paraformaldehyde overnight at 4°C, embedded in a paraffin blocks, and cut into 5-μm-thick sections. The sections were then mounted on slides, deparaffinized, and rehydrated through a graded series of ethanol concentrations. After rehydration, antigen retrieval was performed by boiling sections for 30 min using a Retrieve-All Antigen unmasking system 1 (pH 8; BioLegend, San Diego, CA, USA). Sections were permeabilized with 1% sodium dodecyl sulfate for 10 min at room temperature. mTECs and trachea samples incubated in blocking buffer containing 10% donkey serum and 1% bovine serum albumin for 1 h at room temperature. Samples were incubated overnight at 4°C with primary antibodies diluted in blocking buffer. After washes with PBS, samples were incubated with secondary antibodies and 4′,6-diamidino-2-phenylindole for 30 min at room temperature, washed, and covered with mounting medium and cover slips. Images were acquired using an SP5X laser scanning microscope (Leica) or LSM 700 microscope (Carl Zeiss).

### mTEC cultures

mTECs were isolated from *Zmynd10*^*+/+*^ and *Zmynd10*^*−/−*^ mice at P14 as previously described [39]. Briefly, cells were isolated by overnight digestion with pronase (Roche Diagnostics, Indianapolis, IN, USA) at 4°C and then separated from contaminating fibroblasts by incubation in mTEC basal medium on a Primaria cell culture plate (Corning Inc., Corning, NY, USA) for 3–4 h. mTECs were seeded in collagen-coated apical chambers of transwell permeable supports (0.4-μm polyester membrane; Corning Inc.). Proliferation medium was applied to the apical and basal chambers of the wells, and cells were cultured at 37°C in 5% CO_2_ [40]. For ALI culture conditions, the medium was removed from the apical chamber when mTECs became confluent, and differentiation medium was added to the basal chamber.

### RNA sequencing

Total RNA was isolated from the brain (P14), lung (P14), and testis (P21) tissues obtained from *Zmynd10*^*+/+*^ and *Zmynd10*^*−/−*^ mice using a Qiagen RNA extraction kit (Qiagen, Valencia, CA, USA). RNA sequencing was performed by Theragen Etex (Suwon, Korea). Libraries were constructed with a TruSeq RNA Library Sample Prep kit (Illumina, San Diego, CA, USA), and the enriched library was sequenced on an Illumina HiSeq 2500 system. Sequence reads were mapped against the mouse reference genome (NCBI GRCm38/mm10) and analyzed using CLC Genomics Workbench v.9.0.1 software (CLC Bio, Cambridge, MA, USA).

### Cell culture and transfection

HEK 293T cells were maintained in DMEM supplemented with 10% FBS and penicillin (50 IU/mL)/streptomycin (50 μg/mL). The cells were transfected with plasmids using Lipofectamine PLUS reagent (Invitrogen).

### Immunoblotting, immunoprecipitation, and GST pulldown assays

Experiments were performed as previously described [41]. Immunoblotting was quantified by densitometry using ImageJ software (National Institutes of Health, Bethesda, MD, USA). Immunoprecipitation was performed using EZview Red anti-FLAG M2 or anti-c-Myc affinity gels (Sigma-Aldrich). Pulldown assays with GST-ZMYND10 and GST-MYND were performed as previously described [20].

### Protein stability assay

Cycloheximide chase was used to assess the stability of LRRC6, DNAI1, and DNAI2. HEK293T cells were transfected with Myc-tagged LRRC6, DNAI1, or DNAI2 with or without FLAG-tagged ZMYND10; at 24 h post-transfection, cells were treated with 100 μg/mL cycloheximide (C4859; Sigma-Aldrich) to inhibit new protein synthesis. Cells were harvested at predetermined time points, and LRRC6, DNAI1, and DNAI2 levels were detected by western blotting.

### Statistical analysis

Results are presented as means ± standard errors or standard deviations for the indicated number of experiments. Statistical analysis of continuous data was performed with two-tailed Student’s *t*-test or one-way analysis of variance, with Dunnet’s, Bonferroni, or Dunn post hoc test, as appropriate. Results with *P* values of less than 0.05 were considered statistically significant.

## Supporting information

S1 Fig Generation and validation of *Zmynd10-*targeted alleles. **(A)** Diagram of *Zmynd10*-targeted alleles.
**(B)** Wild-type, heterozygous, and homozygous sex-matched littermates were genotyped by PCR based on the presence of the neomycin cassette, exon 2, exon 3, or exon 4. The wild-type allele was 529 bp, and the mutant allele was 764 bp.
**(C–F)** X-gal staining of lungs (C and D) and testes (E and F) of *Zmynd10* wild-type and heterozygote mice. Each section was counterstained with nuclear fast red. X-gal staining in *Zmynd10*^*+/-*^ lung sections confirms *Zmynd10* expression in the bronchiole. Scale bars, 200 μm. (E and F) X-gal staining revealed that *Zmynd10* was distributed in spermatocytes to spermatids in *Zmynd10*^*+/-*^ testis. Scale bars, 100 μm.
(TIF)

S2 Fig Anti-ZMYND10 antibody generation and validation. **(A)** Western blot analysis of HEK 293T cells transfected with FLAG-ZMYND10 and mouse testis lysates. For each well, 50 μg protein from the testis of *Zmynd10*^*+/+*^ and *Zmynd10*^*-/-*^ mice was used for western blot analysis. ZMYND10 was located at approximately 50 kDa.
**(B and C)** Immunofluorescence analysis of the tracheal epithelium in *Zmynd10*^*+/+*^ (B) and *Zmynd10*^*-/-*^ (C) mice. ZMYND10 (red) was localized in the apical membrane and not in cilia labeled with acetylated α-tubulin (Ac-α-tub, green) in the tracheas of *Zmynd10*^*+/+*^ mice (B). However, ZMYND10 was completely absent from *Zmynd10*^*-/-*^ mice (C). Scale bars, 10 μm.
**(D and E)** Immunofluorescence of acetylated-α-tubulin, γ-tubulin (γ-tub, red), and ZYMND10 in the tracheal epithelium of *Zmynd10*^*+/+*^ mice. ZMYND10 did not colocalize with γ-tubulin.
(TIF)

S3 Fig *Zmynd10*^-/-^ mice were runts and developed hydrocephalus and/or *situs inversus*. **(A and B)** Mouse growth. (A) Photographs of *Zmynd10* wild-type and homozygous mice. Z*mynd10*^*-/-*^ mice were notably smaller than *Zmynd10*^*+/+*^ siblings. (B) Body weights were quantified at 10 days of age.
**(C)** Survival graph of the indicated genotypes and numbers (n).
**(D)** Lungs extracted from a P29-old *Zmynd10*^-/-^ mouse. Lobular structures were completed deteriorated and alveolar spaces were collapsed.
**(E and F)** Consistent with randomization of left-right body asymmetry, *Zmynd10*^-/-^ mice showed *situs inversus* (F). The locations of stomach, heart, liver and spleen are indicated. H, heart; K, kidney; Lu, lung; Sp, spleen; St, stomach.
(TIF)

S4 Fig Inflammation observed in the tracheas of *Zmynd10*^-/-^ mice. TEM images of tracheal epithelia. Both mice generated cilia normally. In contrast to *Zmynd10*^+/+^ mice (A), *Zmynd10*^-/-^ mice (B) exhibited cellular debris in some parts of the tracheal epithelia. Black and white arrowheads are basal bodies and ciliary axonemes, respectively. Black brackets indicate layers of cellular debris and mucus. Scale bar, 500 μm.
(TIF)

S5 Fig Individual channel images of immunofluorescence corresponding to Fig 2K and 2L. mTEC cultures at ALI day 14 were stained with acetylated-α-tubulin (Ac-α-tub, green) and DNAI2 (red). DNAI2 did not colocalize with acetylated α-tubulin and was significantly decreased in *Zmynd10*^*−/−*^ mTECs (B), suggesting that motile cilia lacked ODAs. Scale bar, 10 μm.
(TIF)

S6 Fig Enrichment analysis of gene ontology (GO). GO terms of 77 significantly increased (A) and 76 significantly reduced (B) genes in the testes of *Zmynd10*^-/-^ mice compared with those in *Zmynd10*^+/+^ mice. Gene ontology categories representing molecular function (MF), cellular component (CC), and biological process (BP) were separately analyzed for enrichment. Five of the most significantly enriched gene ontology terms in three categories were plotted against–log (*p* value). t-test, *p* value < 0.05.
(TIF)

S7 Fig ZMYND10 interacted with cytoplasmic proteins. **(A–L)** Interactions between FLAG-tagged ZMYND10 and Myc-tagged IQUB (A and B), Myc-TCTEX1D1 (C and D), DYX1C1-V5 (E and F), Myc-C21ORF59 (G and H), Myc-DNAI1 (I and J), or Myc-DNAI2 (K and L). All constructs were cotransfected into HEK 293T cells and co-immunoprecipitated with anti-FLAG antibodies (A, C, E, G, I, and K) or anti-Myc antibodies (B, D, F, H, J, and L). The immunoblots indicated that antibodies did not show nonspecific bands. Immunoprecipitation showed protein-protein interactions between ZMYND10 and IQUB, TCTEX1D1, DYX1C1, C21ORF59, and DNAI1 (A–J). However, ZMYND10 did not interact with DNAI2 (K and L).
(TIF)

S8 Fig ZMYND10 interacted with HSC70. **(A and B)** Myc-HSC70 and FLAG-ZMYND10 were cotransfected into HEK 293T cells and co-immunoprecipitated with anti-FLAG antibodies (A) or anti-Myc antibodies (B).
**(C)** FLAG-ZMYND10 was transfected into HEK 293T cells, co-immunoprecipitated with anti-FLAG antibodies, and blotted with anti-HSC70 antibodies.
(TIF)

S9 Fig Individual channel images of immunofluorescence corresponding to Fig 4C–4E. mTEC cultures at ALI day 14 were stained for DNAI2 and C21ORF59 (A), acetylated α-tubulin (Ac-α-tub) and C21ORF59 (B), and Ac-α-tub and REPTIN (C). REPTIN and C21ORF59, both of which interact with ZMYND10, were significantly decreased in *Zmynd10*^*−/−*^ mTECs. Scale bar, 10 μm.
(TIF)

S10 Fig The MYND domain of ZMYND10 was necessary for the stabilizing effect of the protein. **(A)** Representative immunoblots of stability assays. Note that ZMYND10 stabilized DNAI1, whereas ZMYND10-p.Gln366*, which lacked the MYND domain, failed to stabilize DNAI1.
**(B)**Graph of band intensities. The graph is summarized from triplicate experiments, and the band intensities were normalized to β-actin levels.
**(C)** Co-immunoprecipitation showing that ZMYND10-pGln366* did not interact with DNAI1, indicating that the MYND domain was necessary for the interaction.
(TIF)

S11 Fig DNAI2 was not stabilized by co-expression of ZMYND10. The stability of DNAI2 was examined by protein stability assays in HEK 293T cells. After treatment with cycloheximide (100 μg/mL), protein samples were harvested at the indicated times.
**(A)** Representative immunoblots. Note that protein levels of DNAI2 were not affected by ZMYND10 co-expression.
**(B)** Graph of band intensities. The graph was summarized from triplicate experiments, and the band intensities were normalized to β-actin levels.
(TIF)

S12 Fig All experiments reflecting the graph corresponding to Fig 6E and 6F. The stability of DNAI2 proteins was examined with protein stability assays. Protein samples were harvested at the indicated times after treatment with cycloheximide (100 μg/mL). All four experiments are summarized in Fig 6F.
(TIF)

S13 Fig Function of ZMYND10 in the cytoplasmic pre-assembly of dynein arms. **(A)**ZMYND10 binds to and stabilizes DNAI1. DNAI1 forms a complex with DNAI2, and heavy chain proteins are then attached to the intermediate chain complex. ZMYND10 may also regulate proper folding of DNAI1 or the assembly of the intermediate chain complex.
**(B)**In the absence of ZMYND10, both DNAI1 and DNAI2 are unstable and degraded.
(TIF)

S14 Fig Uncropped images of western blot data. (TIF)

S1 Movie Videomicroscopy of brain ventricles from a P16 wild-type mouse. (MP4)

S2 Movie Videomicroscopy of brain ventricles from a P16 *Zmynd10*^*-/-*^ mouse. (MP4)

S1 Table Variation between initial and final frequencies of aedes aegypti NaV alleles from São Paulo populations. (XLSX)
