# Supplementary figures and images for: ZMYND10 stabilizes intermediate chain proteins in the cytoplasmic pre-assembly of dynein arms
Source: PLoS Genet. 2018 Mar 30;14(3):e1007316. doi: 10.1371/journal.pgen.1007316 (PMC5895051; doi:10.1371/journal.pgen.1007316)

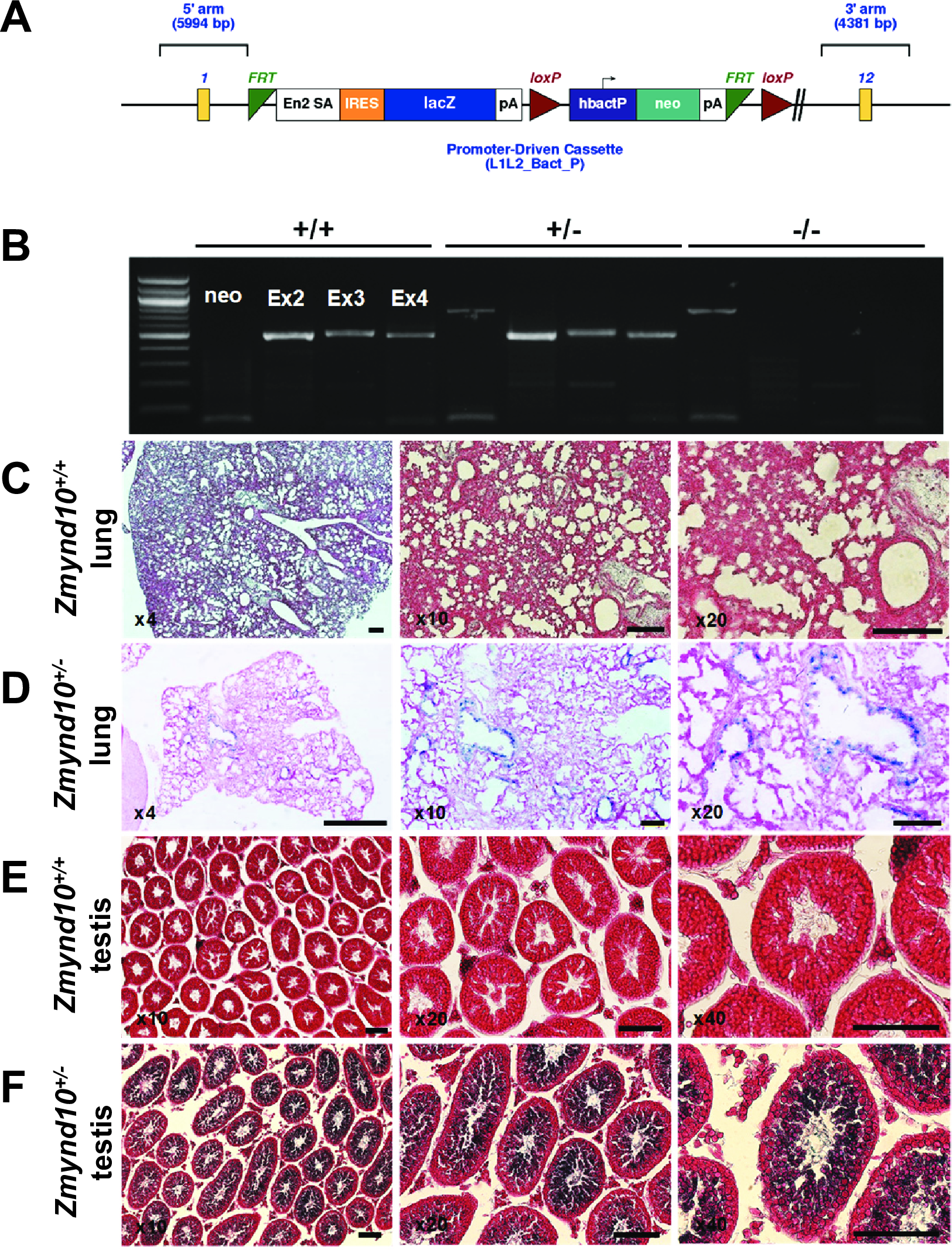

Supplement: S1 Fig — (A) Diagram of Zmynd10-targeted alleles. (B) Wild-type, heterozygous, and homozygous sex-matched littermates were genotyped by PCR based on the presence of the neomycin cassette, exon 2, exon 3, or exon 4. The wild-type allele was 529 bp, and the mutant allele was 764 bp. (C–F) X-gal staining of lungs (C and D) and testes (E and F) of Zmynd10 wild-type and heterozygote mice. Each section was counterstained with nuclear fast red. X-gal staining in Zmynd10+/- lung sections confirms Zmynd10 expression in the bronchiole. Scale bars, 200 μm. (E and F) X-gal staining revealed that Zmynd10 was distributed in spermatocytes to spermatids in Zmynd10+/- testis. Scale bars, 100 μm. (TIF) [file pgen.1007316.s001.tif]

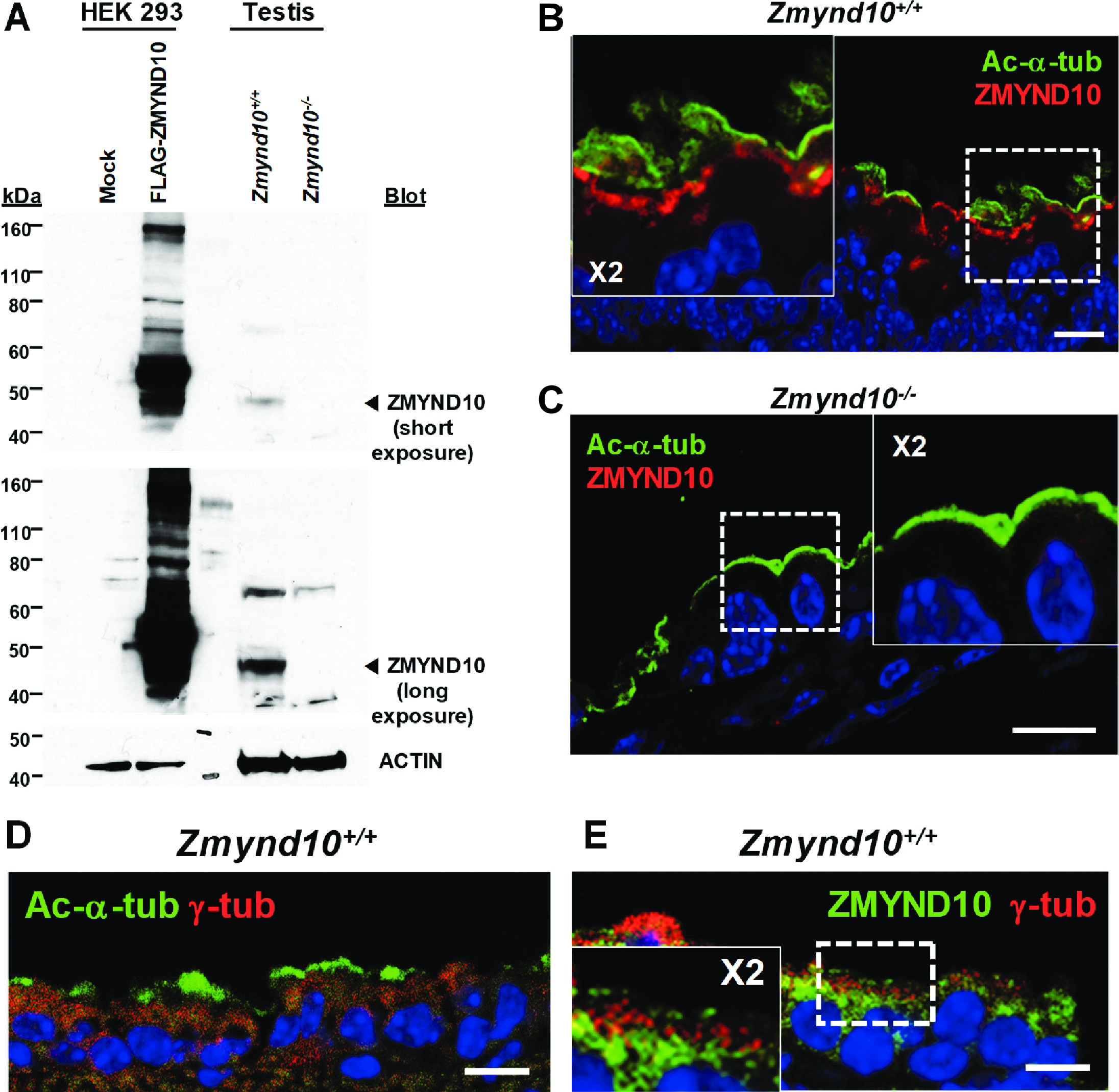

Supplement: S2 Fig — (A) Western blot analysis of HEK 293T cells transfected with FLAG-ZMYND10 and mouse testis lysates. For each well, 50 μg protein from the testis of Zmynd10+/+ and Zmynd10-/- mice was used for western blot analysis. ZMYND10 was located at approximately 50 kDa. (B and C) Immunofluorescence analysis of the tracheal epithelium in Zmynd10+/+ (B) and Zmynd10-/- (C) mice. ZMYND10 (red) was localized in the apical membrane and not in cilia labeled with acetylated α-tubulin (Ac-α-tub, green) in the tracheas of Zmynd10+/+ mice (B). However, ZMYND10 was completely absent from Zmynd10-/- mice (C). Scale bars, 10 μm. (D and E) Immunofluorescence of acetylated-α-tubulin, γ-tubulin (γ-tub, red), and ZYMND10 in the tracheal epithelium of Zmynd10+/+ mice. ZMYND10 did not colocalize with γ-tubulin. (TIF) [file pgen.1007316.s002.tif]

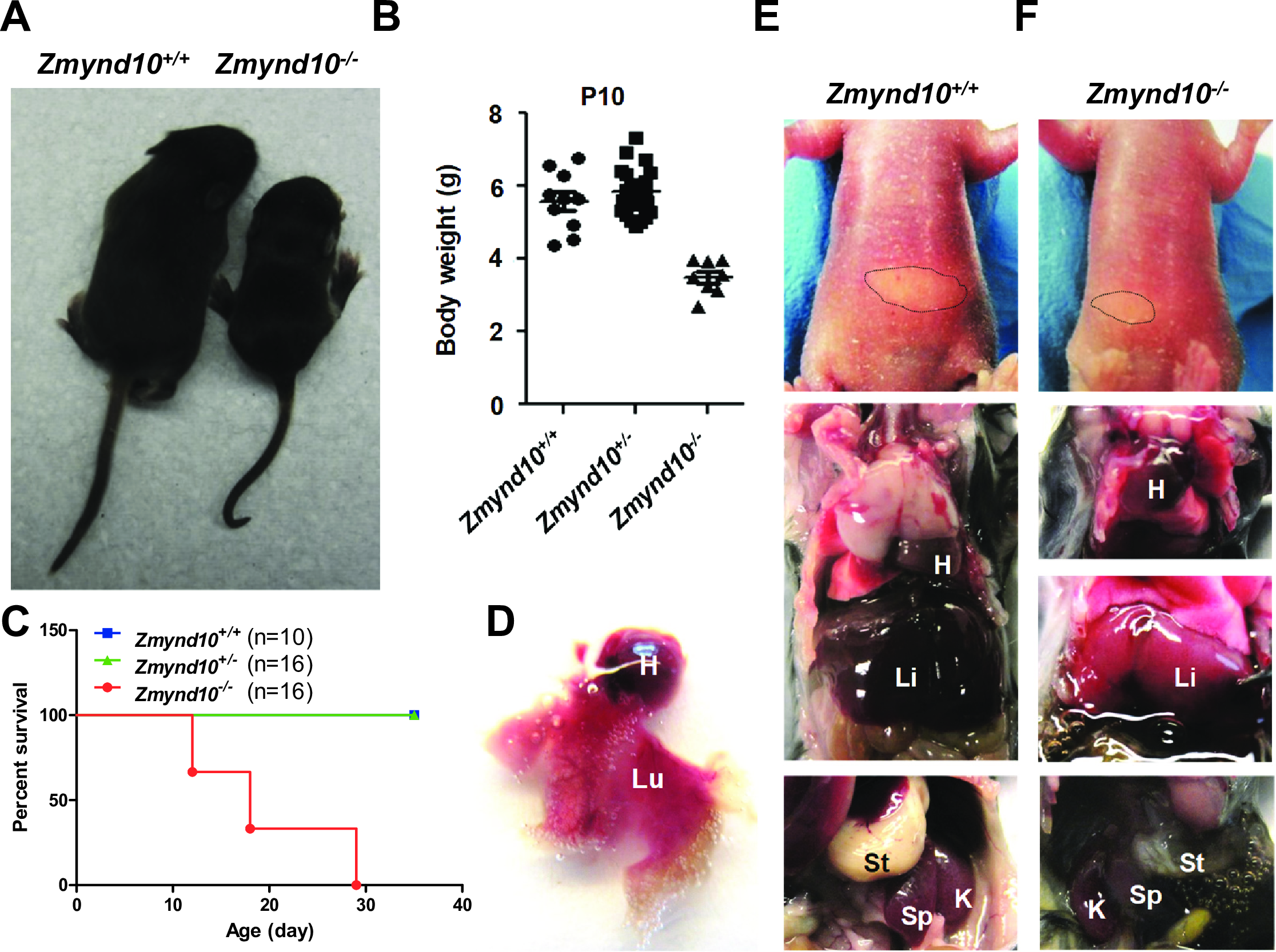

Supplement: S3 Fig — (A and B) Mouse growth. (A) Photographs of Zmynd10 wild-type and homozygous mice. Zmynd10-/- mice were notably smaller than Zmynd10+/+ siblings. (B) Body weights were quantified at 10 days of age. (C) Survival graph of the indicated genotypes and numbers (n). (D) Lungs extracted from a P29-old Zmynd10-/- mouse. Lobular structures were completed deteriorated and alveolar spaces were collapsed. (E and F) Consistent with randomization of left-right body asymmetry, Zmynd10-/- mice showed situs inversus (F). The locations of stomach, heart, liver and spleen are indicated. H, heart; K, kidney; Lu, lung; Sp, spleen; St, stomach. (TIF) [file pgen.1007316.s003.tif]

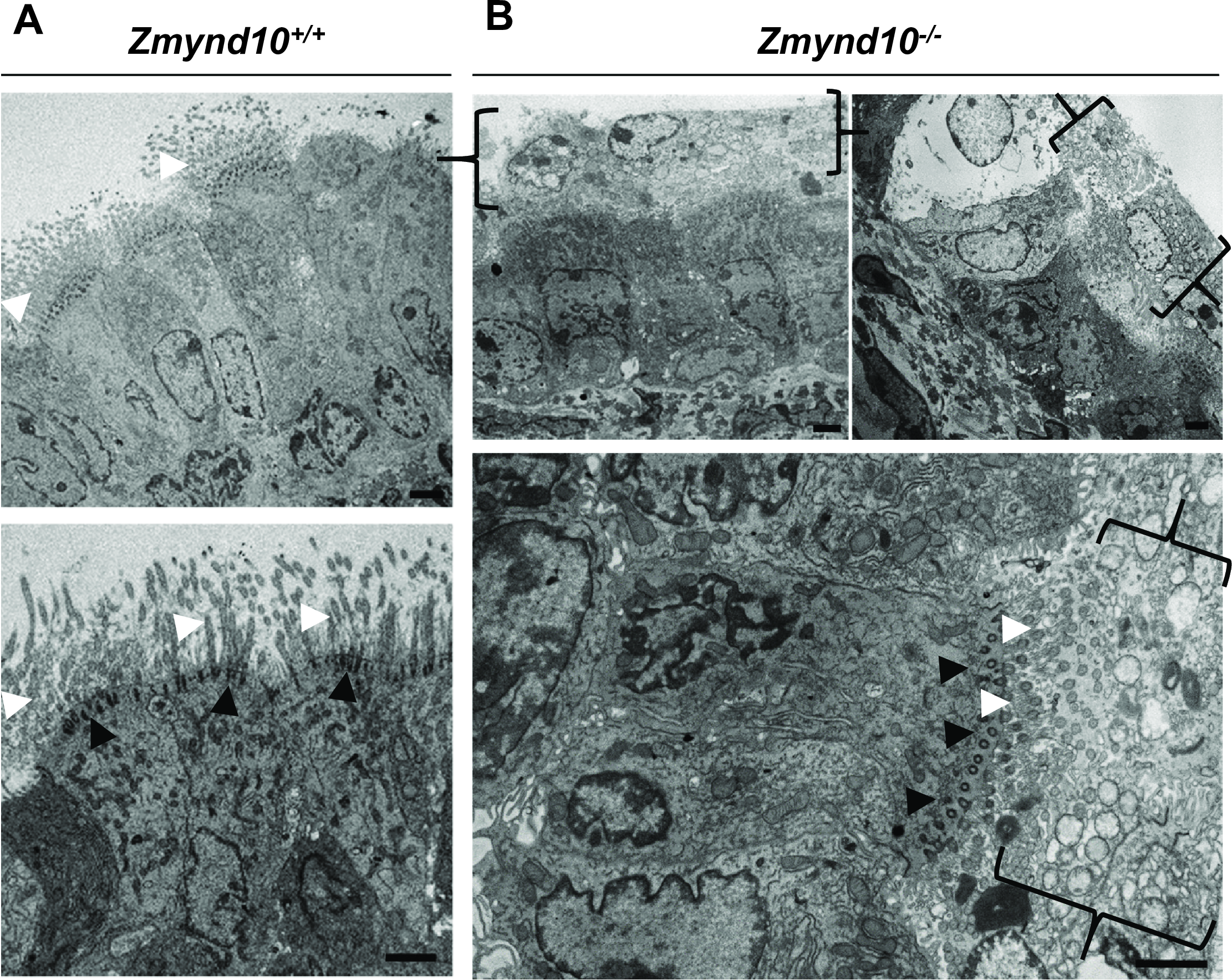

Supplement: S4 Fig — TEM images of tracheal epithelia. Both mice generated cilia normally. In contrast to Zmynd10+/+ mice (A), Zmynd10-/- mice (B) exhibited cellular debris in some parts of the tracheal epithelia. Black and white arrowheads are basal bodies and ciliary axonemes, respectively. Black brackets indicate layers of cellular debris and mucus. Scale bar, 500 μm. (TIF) [file pgen.1007316.s004.tif]

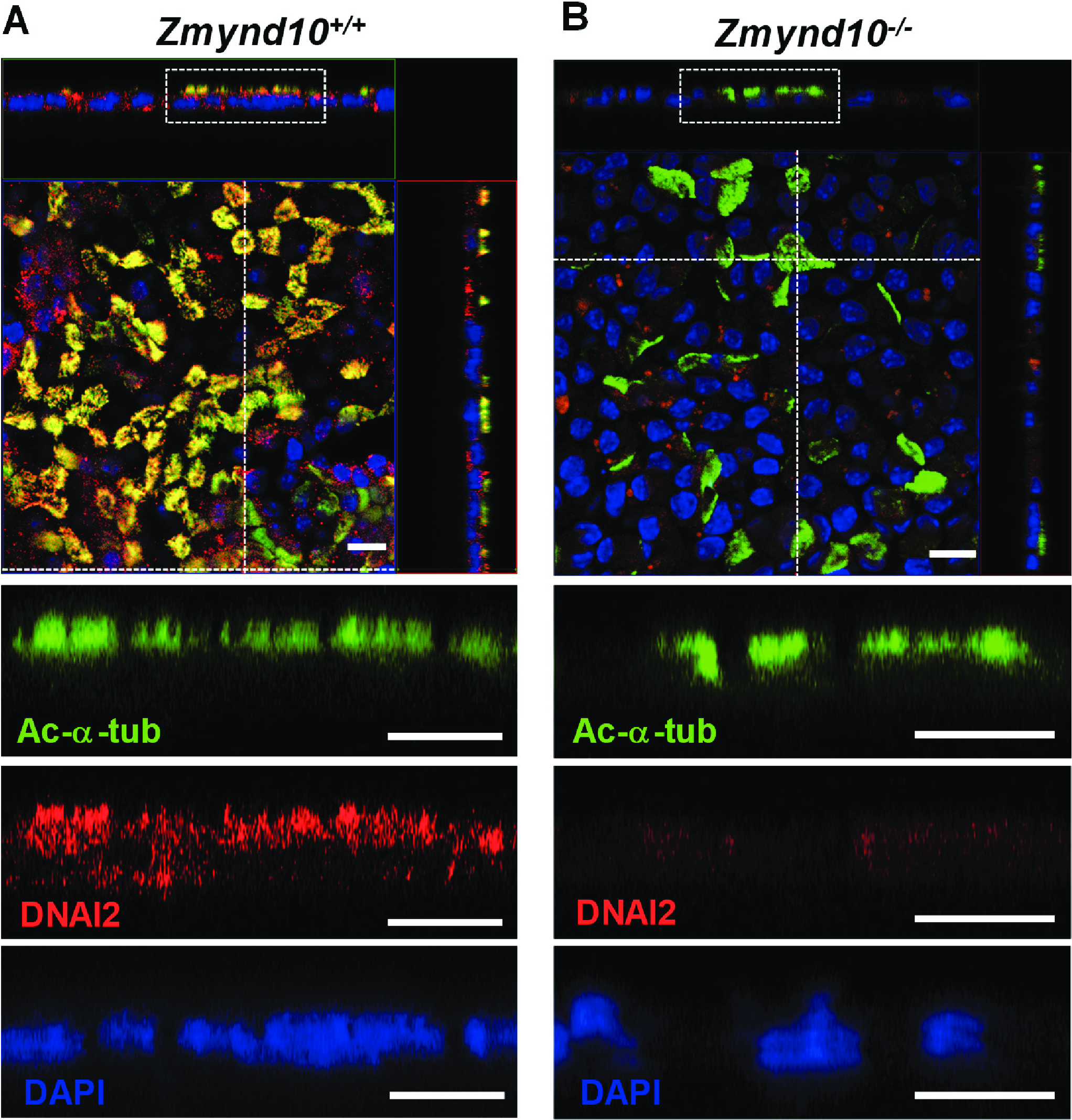

Supplement: S5 Fig — mTEC cultures at ALI day 14 were stained with acetylated-α-tubulin (Ac-α-tub, green) and DNAI2 (red). DNAI2 did not colocalize with acetylated α-tubulin and was significantly decreased in Zmynd10−/− mTECs (B), suggesting that motile cilia lacked ODAs. Scale bar, 10 μm. (TIF) [file pgen.1007316.s005.tif]

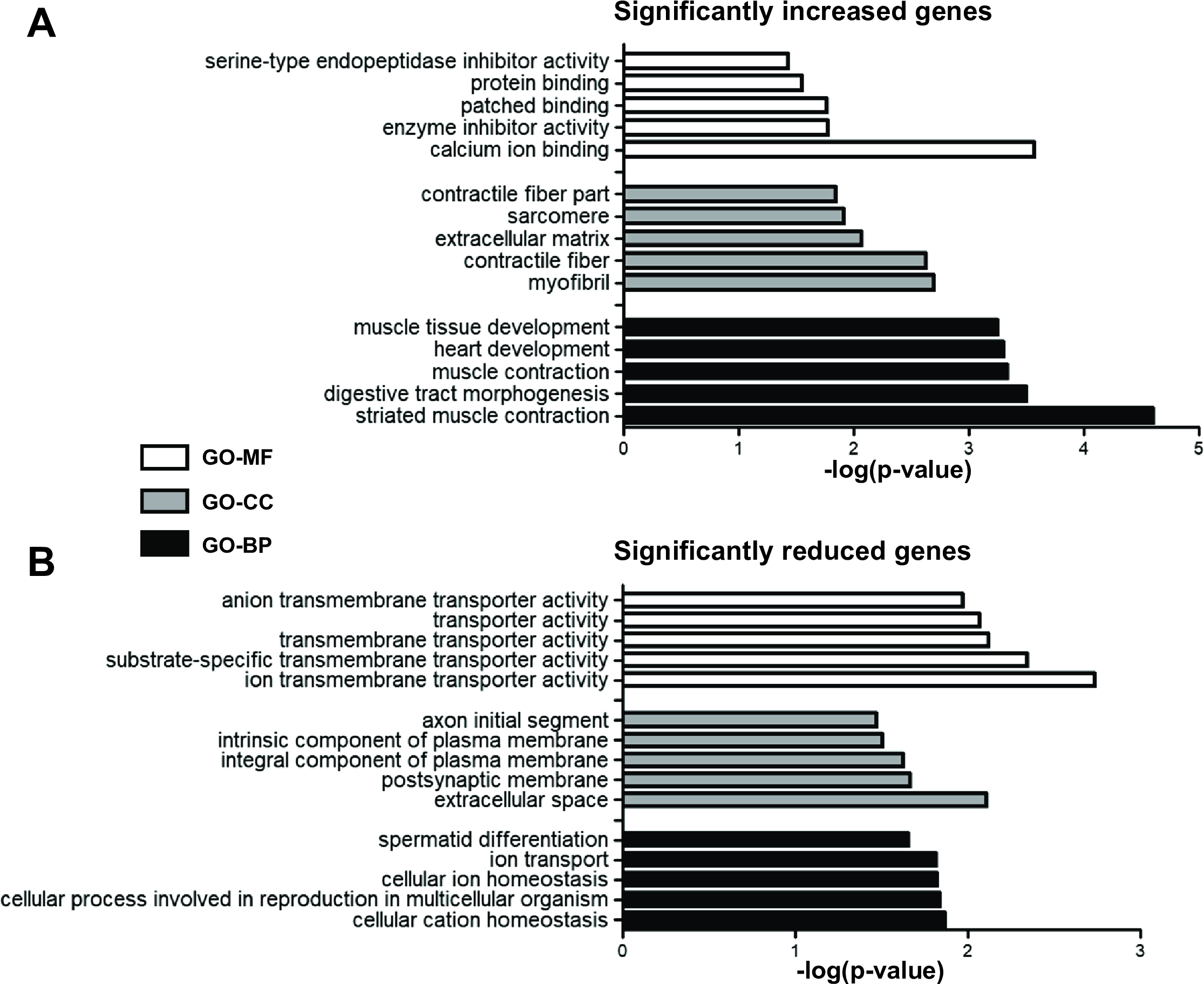

Supplement: S6 Fig — GO terms of 77 significantly increased (A) and 76 significantly reduced (B) genes in the testes of Zmynd10-/- mice compared with those in Zmynd10+/+ mice. Gene ontology categories representing molecular function (MF), cellular component (CC), and biological process (BP) were separately analyzed for enrichment. Five of the most significantly enriched gene ontology terms in three categories were plotted against–log (p value). t-test, p value < 0.05. (TIF) [file pgen.1007316.s006.tif]

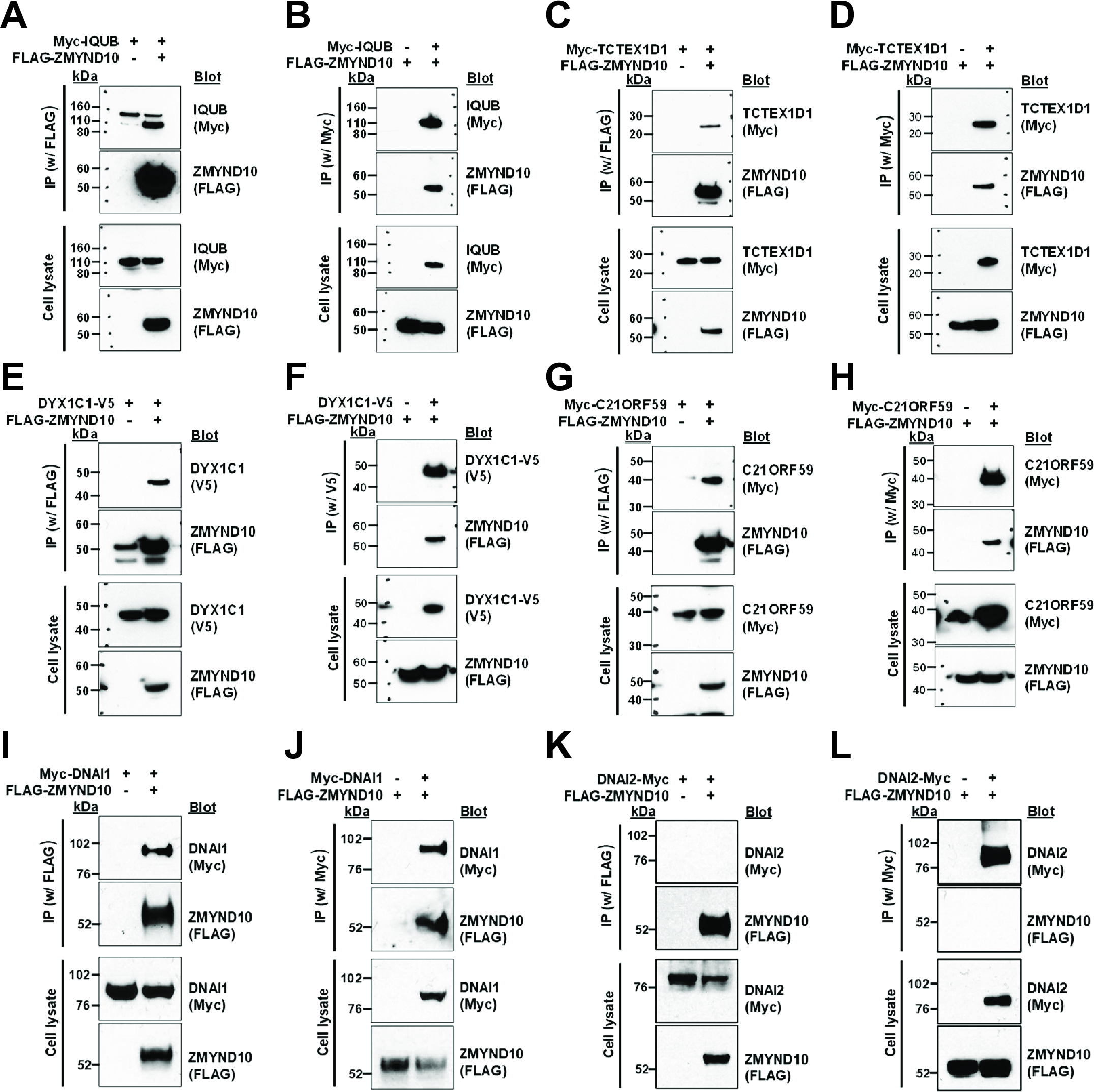

Supplement: S7 Fig — (A–L) Interactions between FLAG-tagged ZMYND10 and Myc-tagged IQUB (A and B), Myc-TCTEX1D1 (C and D), DYX1C1-V5 (E and F), Myc-C21ORF59 (G and H), Myc-DNAI1 (I and J), or Myc-DNAI2 (K and L). All constructs were cotransfected into HEK 293T cells and co-immunoprecipitated with anti-FLAG antibodies (A, C, E, G, I, and K) or anti-Myc antibodies (B, D, F, H, J, and L). The immunoblots indicated that antibodies did not show nonspecific bands. Immunoprecipitation showed protein-protein interactions between ZMYND10 and IQUB, TCTEX1D1, DYX1C1, C21ORF59, and DNAI1 (A–J). However, ZMYND10 did not interact with DNAI2 (K and L). (TIF) [file pgen.1007316.s007.tif]

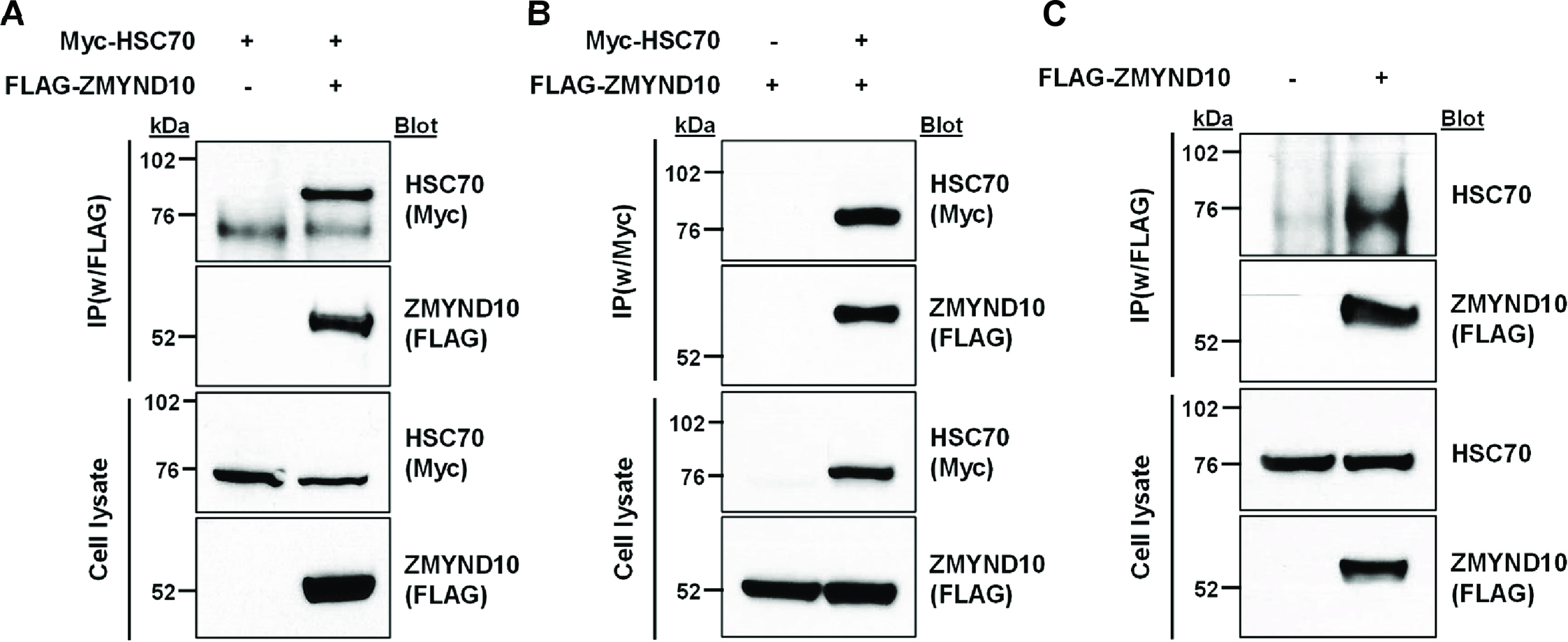

Supplement: S8 Fig — (A and B) Myc-HSC70 and FLAG-ZMYND10 were cotransfected into HEK 293T cells and co-immunoprecipitated with anti-FLAG antibodies (A) or anti-Myc antibodies (B). (C) FLAG-ZMYND10 was transfected into HEK 293T cells, co-immunoprecipitated with anti-FLAG antibodies, and blotted with anti-HSC70 antibodies. (TIF) [file pgen.1007316.s008.tif]

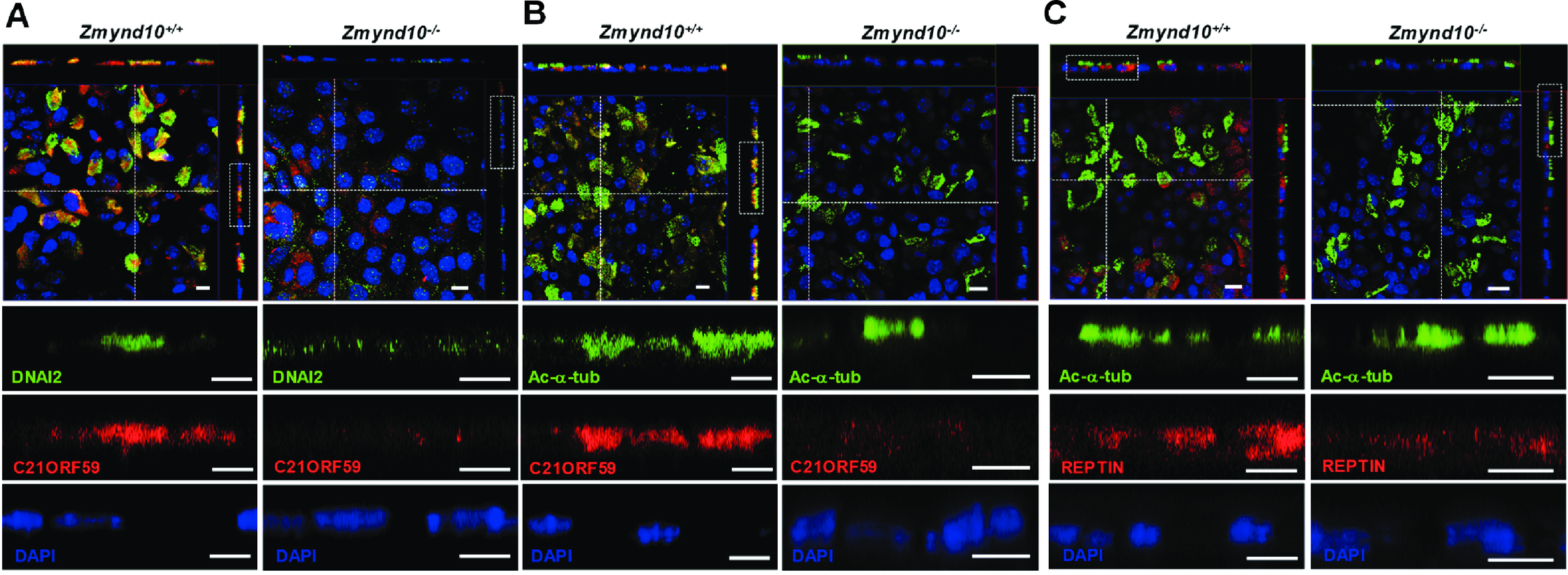

Supplement: S9 Fig — mTEC cultures at ALI day 14 were stained for DNAI2 and C21ORF59 (A), acetylated α-tubulin (Ac-α-tub) and C21ORF59 (B), and Ac-α-tub and REPTIN (C). REPTIN and C21ORF59, both of which interact with ZMYND10, were significantly decreased in Zmynd10−/− mTECs. Scale bar, 10 μm. (TIF) [file pgen.1007316.s009.tif]

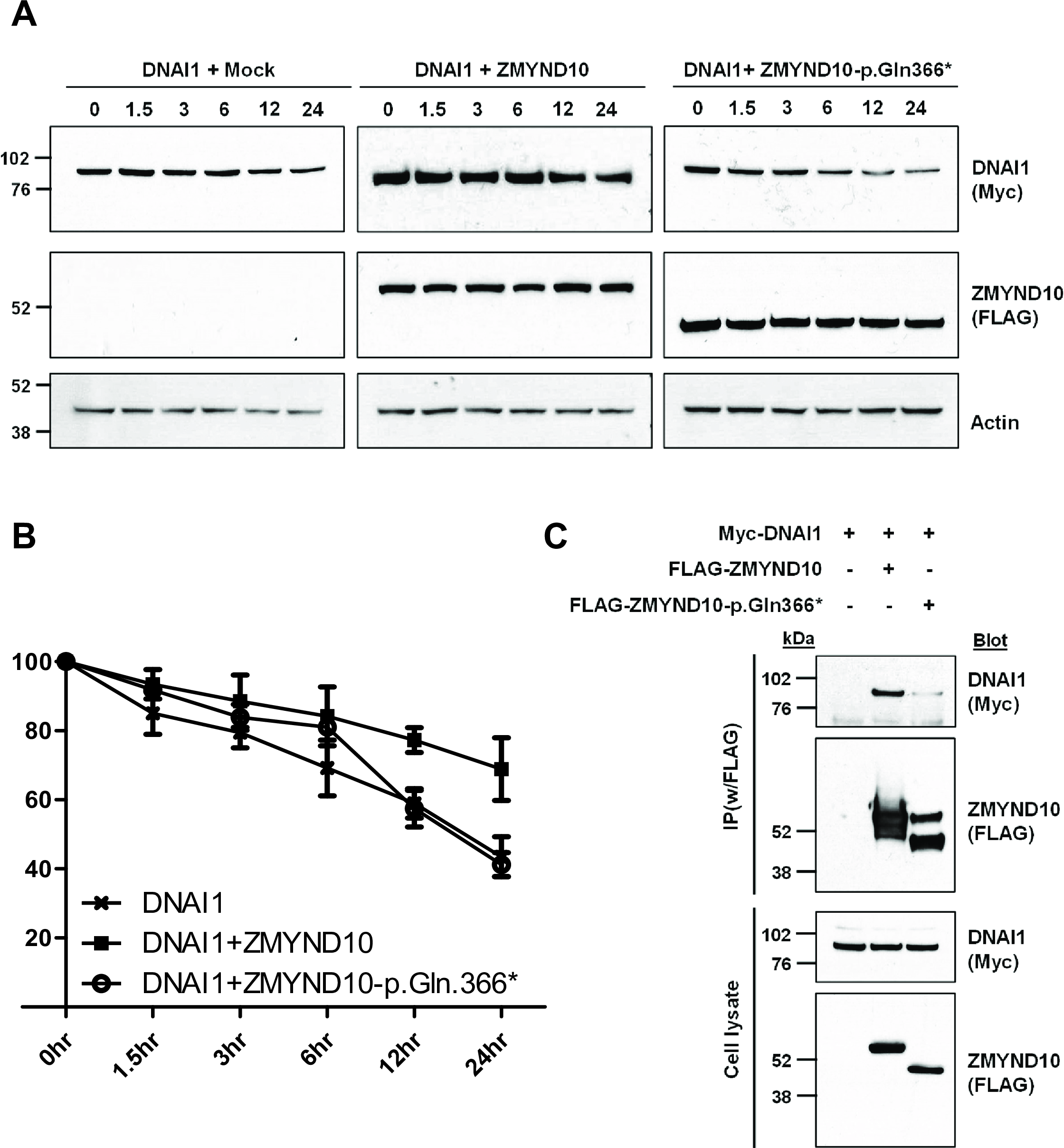

Supplement: S10 Fig — (A) Representative immunoblots of stability assays. Note that ZMYND10 stabilized DNAI1, whereas ZMYND10-p.Gln366*, which lacked the MYND domain, failed to stabilize DNAI1. (B)Graph of band intensities. The graph is summarized from triplicate experiments, and the band intensities were normalized to β-actin levels. (C) Co-immunoprecipitation showing that ZMYND10-pGln366* did not interact with DNAI1, indicating that the MYND domain was necessary for the interaction. (TIF) [file pgen.1007316.s010.tif]

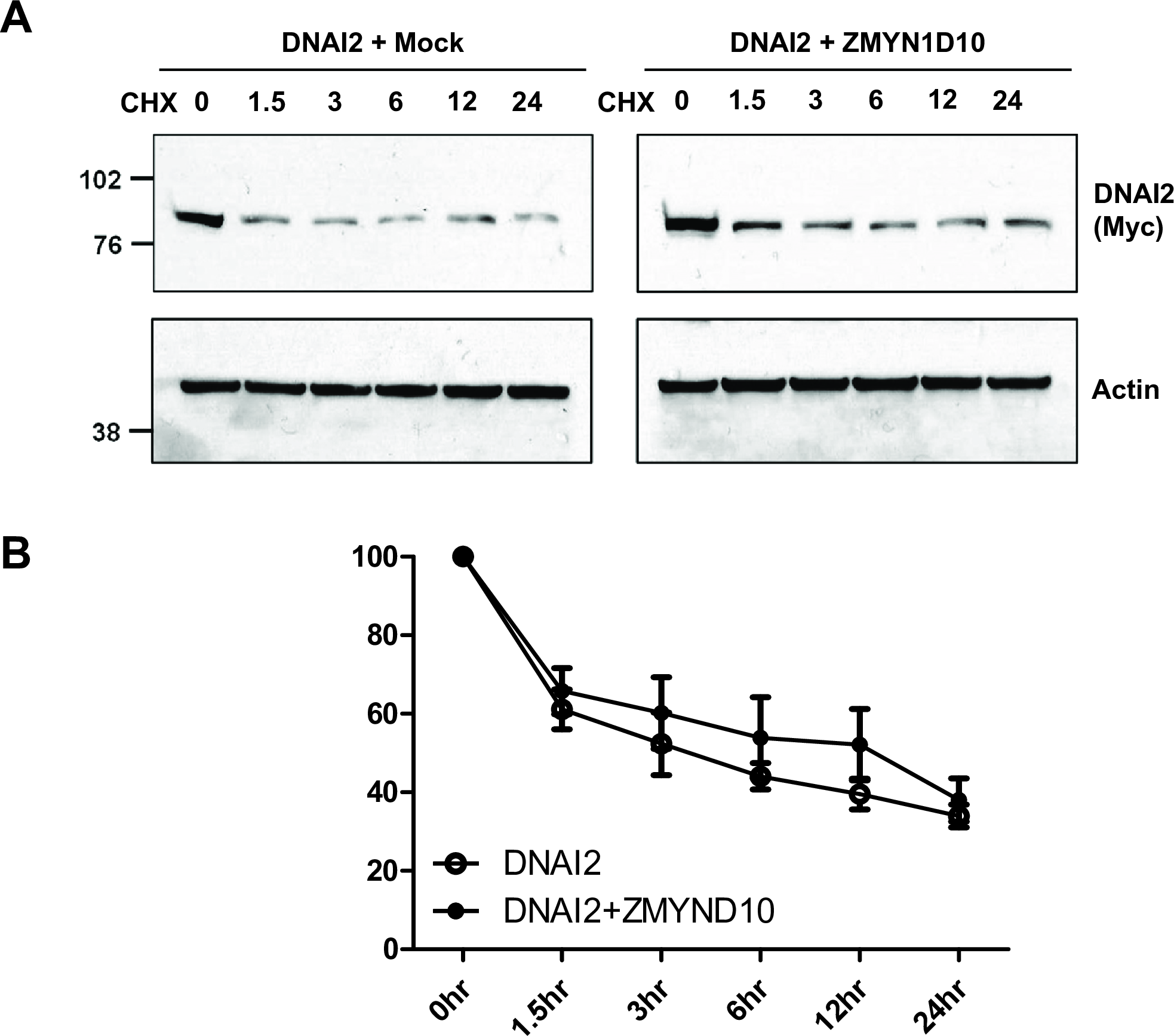

Supplement: S11 Fig — The stability of DNAI2 was examined by protein stability assays in HEK 293T cells. After treatment with cycloheximide (100 μg/mL), protein samples were harvested at the indicated times. (A) Representative immunoblots. Note that protein levels of DNAI2 were not affected by ZMYND10 co-expression. (B) Graph of band intensities. The graph was summarized from triplicate experiments, and the band intensities were normalized to β-actin levels. (TIF) [file pgen.1007316.s011.tif]

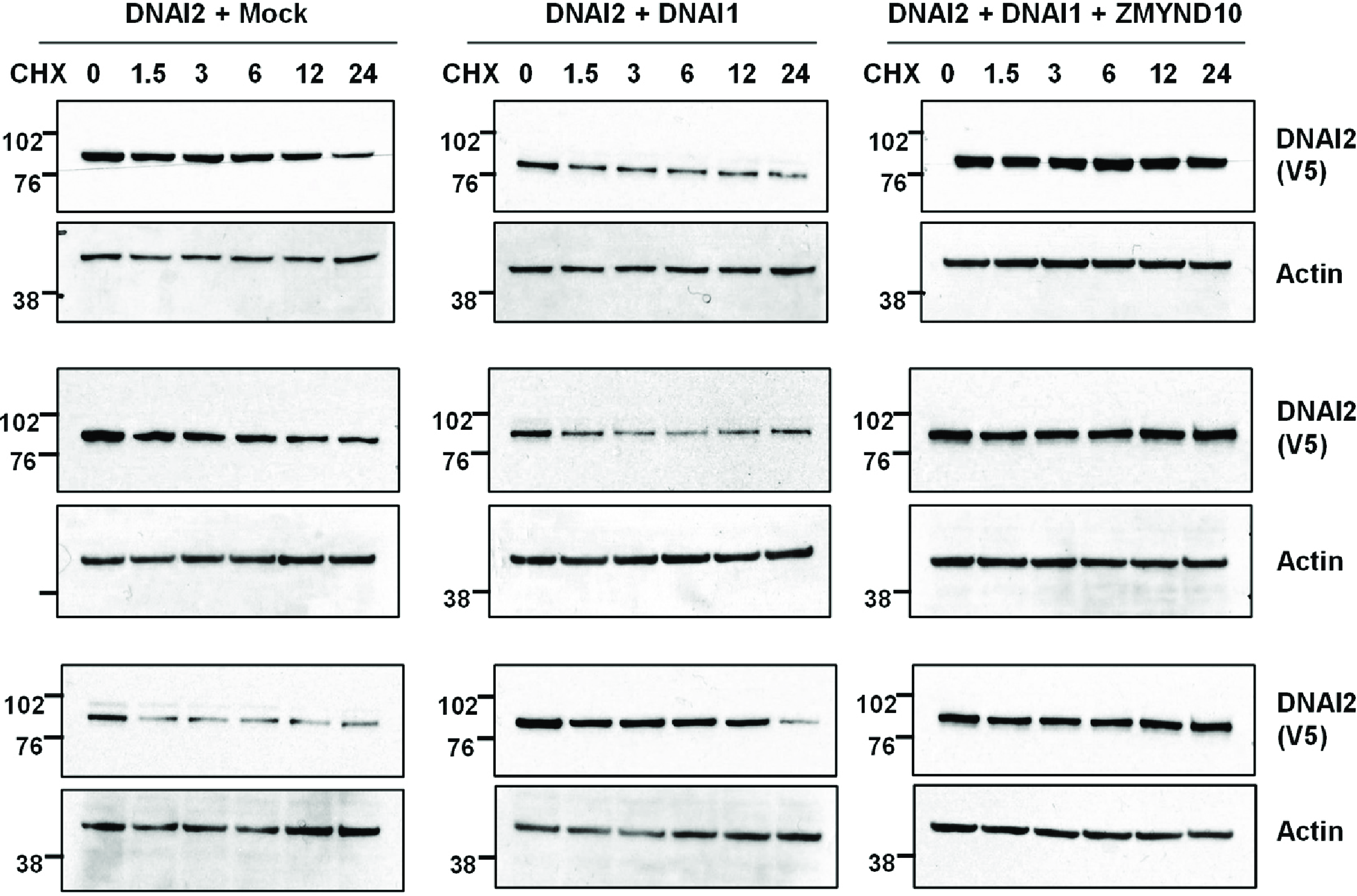

Supplement: S12 Fig — The stability of DNAI2 proteins was examined with protein stability assays. Protein samples were harvested at the indicated times after treatment with cycloheximide (100 μg/mL). All four experiments are summarized in Fig 6F. (TIF) [file pgen.1007316.s012.tif]

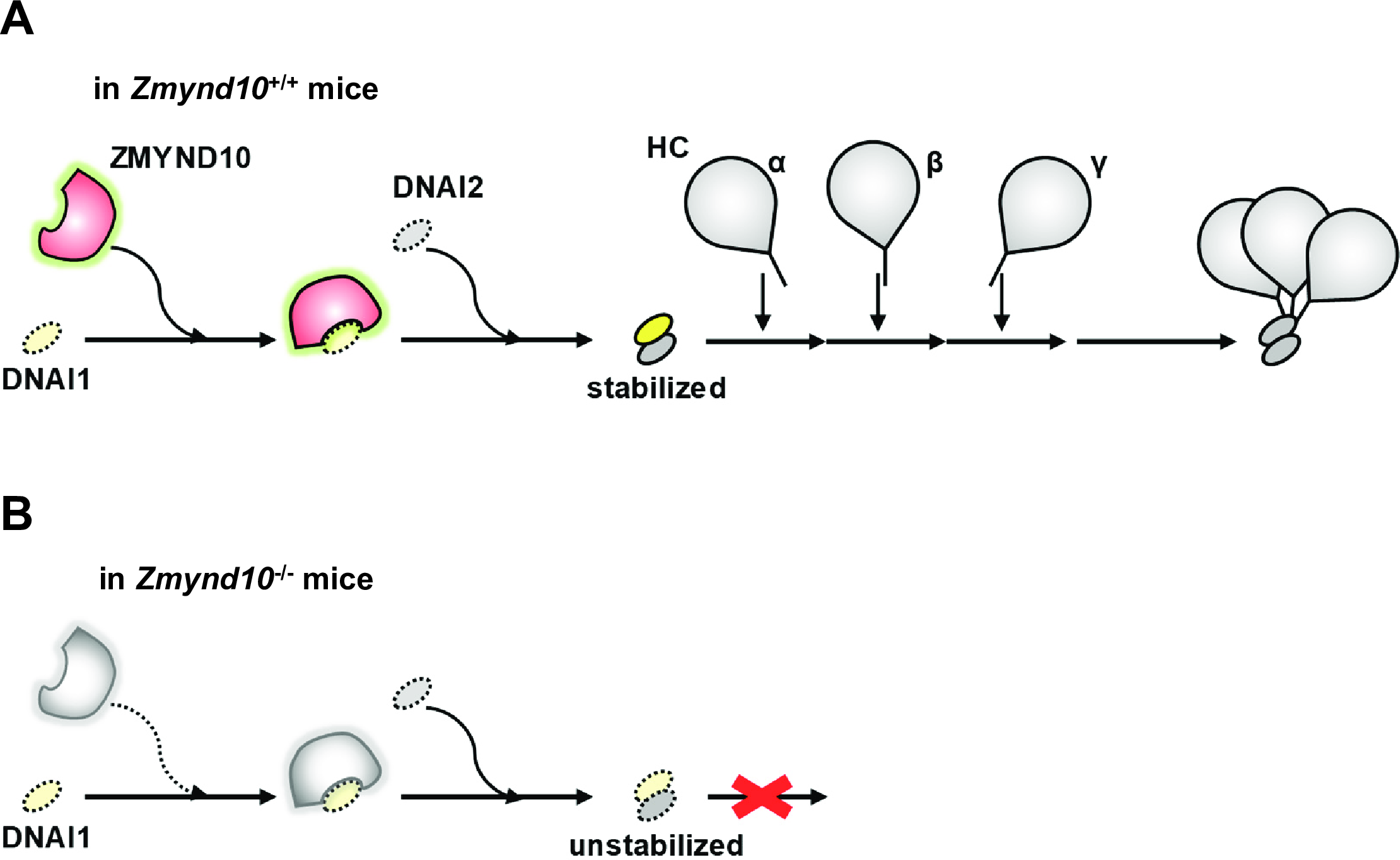

Supplement: S13 Fig — (A)ZMYND10 binds to and stabilizes DNAI1. DNAI1 forms a complex with DNAI2, and heavy chain proteins are then attached to the intermediate chain complex. ZMYND10 may also regulate proper folding of DNAI1 or the assembly of the intermediate chain complex. (B)In the absence of ZMYND10, both DNAI1 and DNAI2 are unstable and degraded. (TIF) [file pgen.1007316.s013.tif]

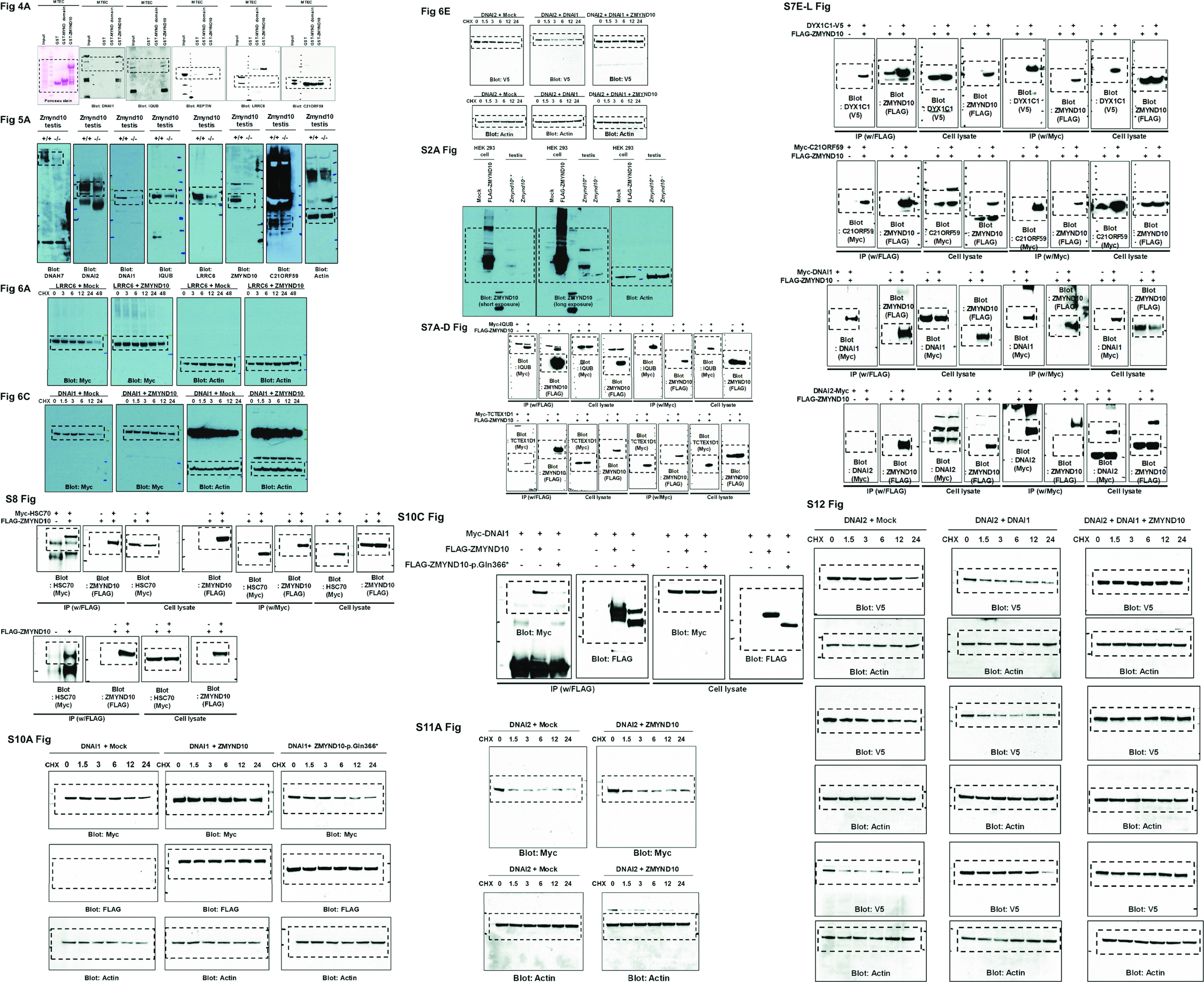

Supplement: S14 Fig — (TIF) [file pgen.1007316.s014.tif]
